# Signaling pathways and targeted therapeutic strategies for polycystic ovary syndrome

**DOI:** 10.3389/fendo.2023.1191759

**Published:** 2023-10-19

**Authors:** Kexin Wang, Yanhua Li

**Affiliations:** ^1^The Second School of Clinical Medicine, Zhejiang Chinese Medical University, Hangzhou, China; ^2^Department of General Practice, The Second Affiliated Hospital of Zhejiang Chinese Medical University, Hangzhou, China

**Keywords:** autophagy, hyperandrogenism, insulin resistance, polycystic ovary syndrome, signaling pathways

## Abstract

Polycystic ovary syndrome (PCOS) is the most common endocrine disorder among women of reproductive age. Although promising strides have been made in the field of PCOS over the past decades, the distinct etiologies of this syndrome are not fully elucidated. Prenatal factors, genetic variation, epigenetic mechanisms, unhealthy lifestyles, and environmental toxins all contribute to the development of this intricate and highly heterogeneous metabolic, endocrine, reproductive, and psychological disorder. Moreover, interactions between androgen excess, insulin resistance, disruption to the hypothalamic–pituitary–ovary (HPO) axis, and obesity only make for a more complex picture. In this review, we investigate and summarize the related molecular mechanisms underlying PCOS pathogenesis from the perspective of the level of signaling pathways, including PI3K/Akt, TGF-β/Smads, Wnt/β-catenin, and Hippo/YAP. Additionally, this review provides an overview of prospective therapies, such as exosome therapy, gene therapy, and drugs based on traditional Chinese medicine (TCM) and natural compounds. By targeting these aberrant pathways, these interventions primarily alleviate inflammation, insulin resistance, androgen excess, and ovarian fibrosis, which are typical symptoms of PCOS. Overall, we hope that this paper will pave the way for better understanding and management of PCOS in the future.

## 1 Introduction

**Figure 1:**
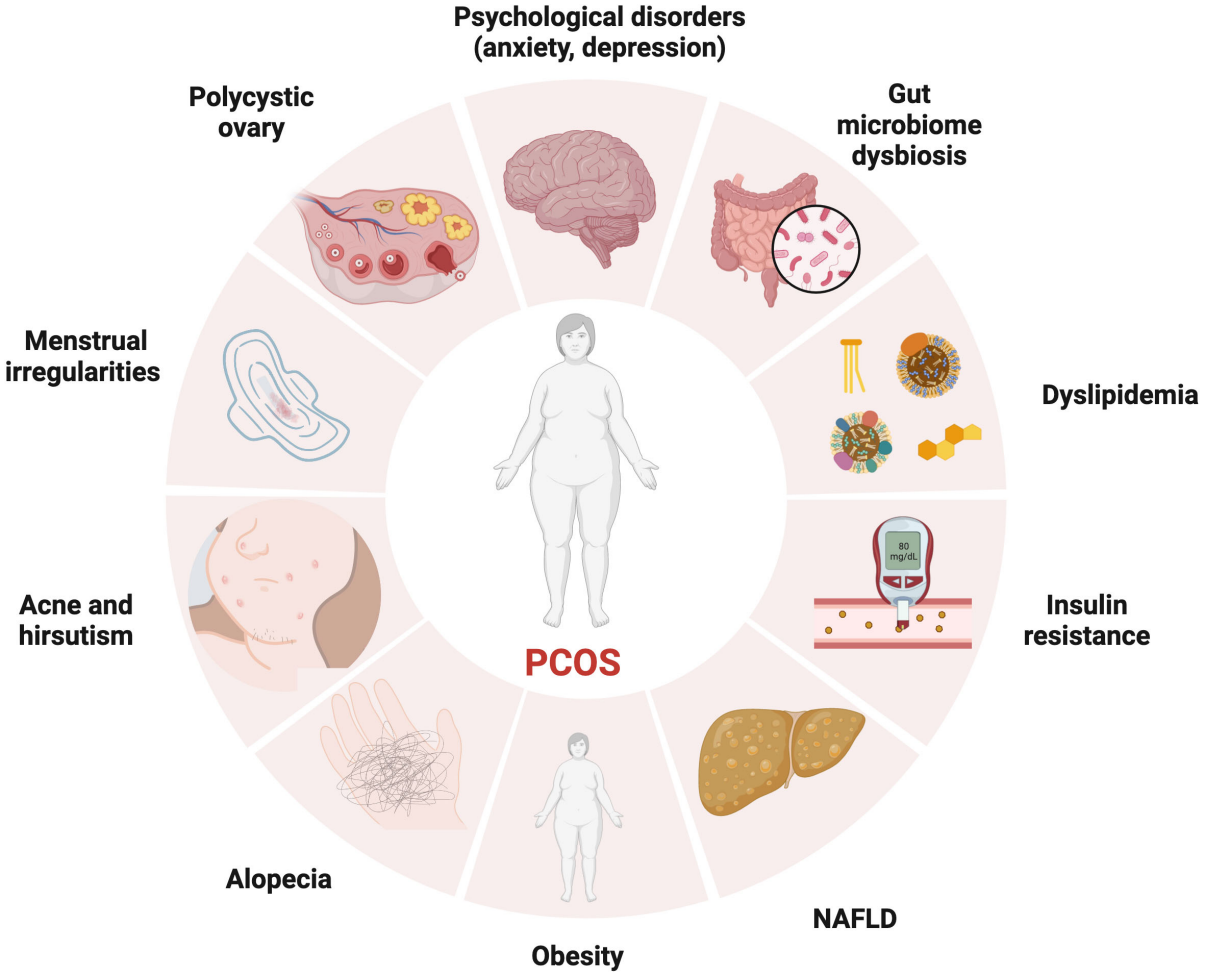
The manifestations of PCOS, a chronic endocrine, metabolic, reproductive, and psychological disorder with various symptoms and signs. It is primarily characterized by ovulation dysfunction (manifesting in the form of menstrual irregularities, such as oligomenorrhea and amenorrhea), hyperandrogenemia (manifesting as hirsutism, acne, or alopecia), and polycystic ovarian morphology.

**Figure 2:**
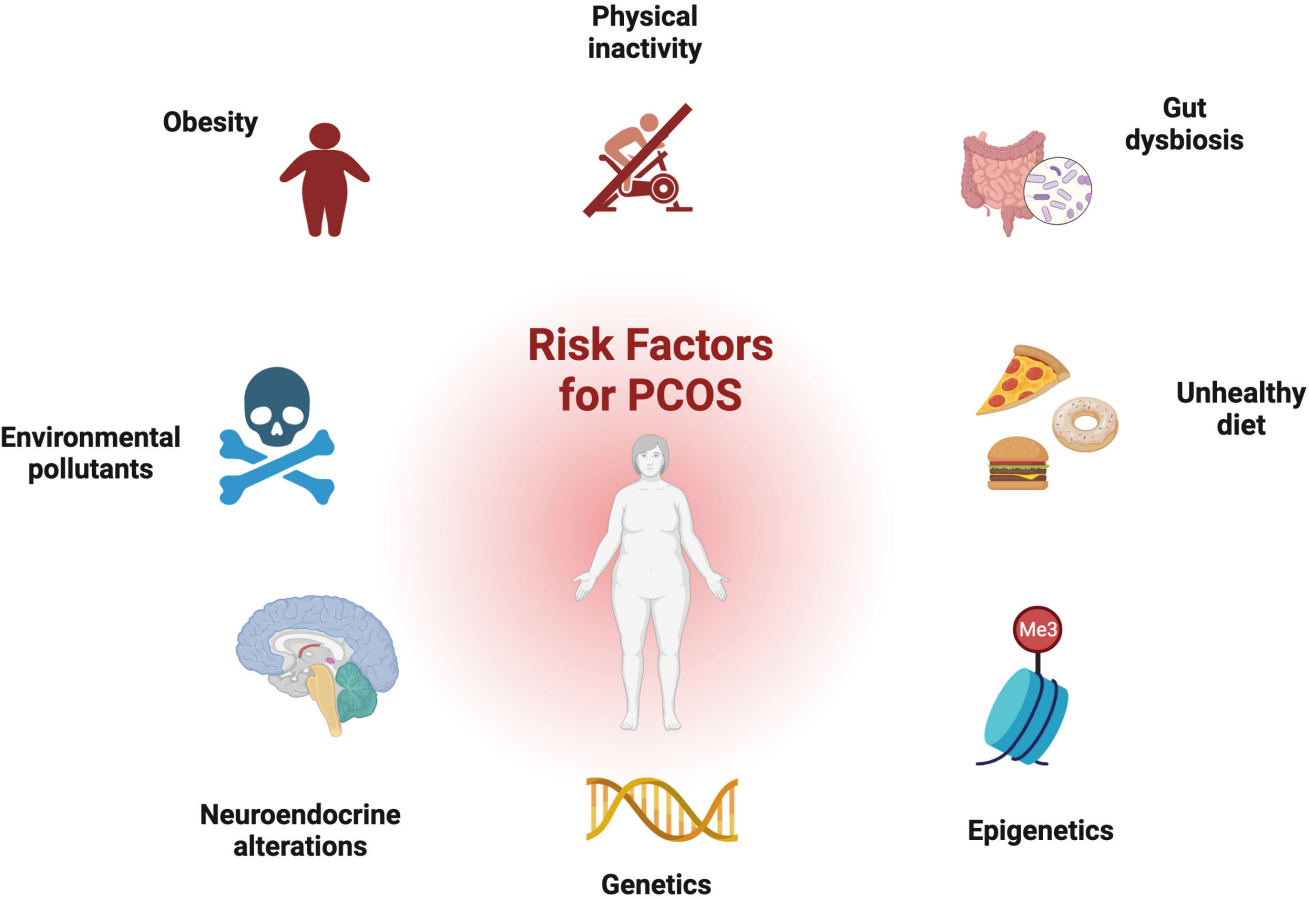
Risk factors for PCOS: genetic and epigenetic factors, disturbance of the hypothalamus–pituitary–ovary (HPO) axis, environmental toxins, dysbiosis of the gut microbiota, and obesity all contribute to development of the disorder.

PCOS is the most common endocrine disorder in women of reproductive age, with an incidence of 6–10% globally, regardless of ethnicity. It is characterized mainly by ovulation dysfunction, hyperandrogenemia (HA), and polycystic ovarian morphology (PCOM). The threshold for PCOM is the presence of more than 20 follicles per ovary and/or an ovarian volume ≥10 ml measured for either ovary via ultrasound (1). According to the Rotterdam criteria, two of the three aforementioned features are required to be present for diagnosis of PCOS. These criteria have been widely endorsed. By contrast, the definition of PCOS from the National Institute of Health (NIH) mainly focuses on two aspects: HA and ovulatory dysfunction (2). Finally, the Androgen Excess Society criteria require the presence of HA along with either ovulatory dysfunction or PCOM, or both. Patients should be carefully evaluated to rule out other conditions that have similar PCOS-like symptoms. Notably, despite the high prevalence of insulin resistance (IR) in PCOS patients, this is not recognized as a diagnostic criterion (3). Based on the three criteria, four different clinical phenotypes are recognized. More recently, two subtypes of PCOS with distinct biochemical characteristics have been identified through phenotypic clustering analysis (4). The first is the reproductive subtype, characterized by higher levels of luteinizing hormone (LH) and sex hormone binding globulin (SHBG), with relatively low BMI and insulin levels. The other subtype is the metabolic subtype, manifesting in the form of higher BMI and insulin levels with relatively low SHBG and LH levels. More active investigation is needed to determine whether these phenotypes reflect the etiology of PCOS and thus can be used to provide tailored treatment for each individual. As PCOS is a multi-factorial and highly heterogeneous endocrine, metabolic, and psychological disorder, its clinical manifestations can be diverse, often leading to delayed diagnosis and misdiagnosis, which can cause long-lasting and distressing complications for patients (**Figures 1**, **2**). Unfortunately, the etiologies of PCOS remain unknown and there is no cure for this condition. In this review, we summarize several key signaling pathways closely involved in the pathogenesis of PCOS: the PI3K/Akt, TLR4/NF-κB, Nrf2/HO-1, AMPK, MAPK, JAK/STAT, Wnt/β-catenin, Notch, Hippo/YAP, TGF-β/Smads, and hedgehog pathways. Additionally, we discuss current therapeutic strategies that target these aberrant signaling pathways.

## 2 Neuroendocrine imbalance

**Figure 3:**
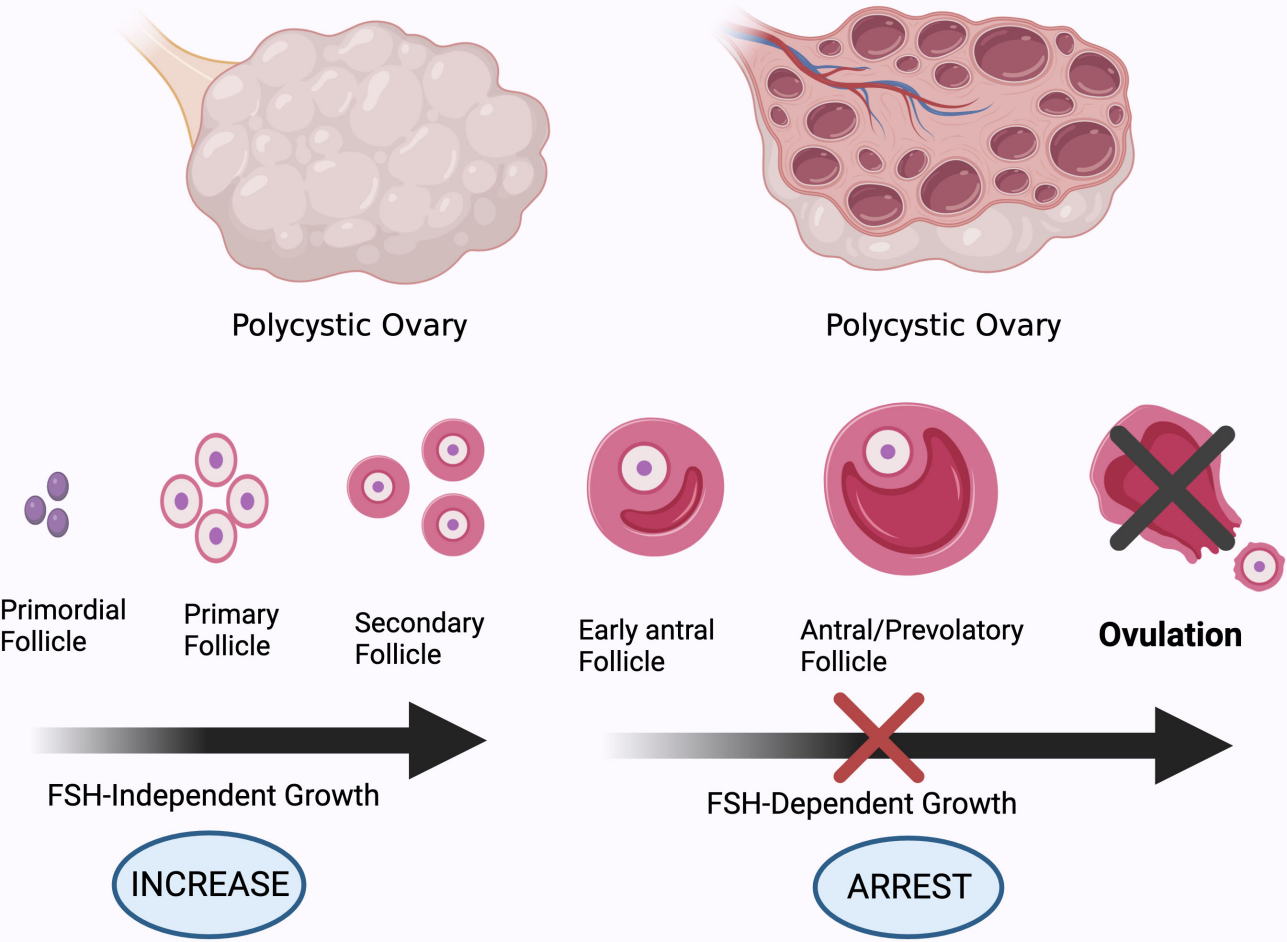
Different stages of development of ovarian follicles. High AMH levels and GnRH pulsatility, and subsequently increased androgen production by theca cells, impair follicle maturation in PCOS patients, resulting in anovulation. Created using BioRender.com.

More recently, attention has been widely paid to the role of the neuroendocrine backdrop in the development of PCOS (5). Simply blocking the androgen receptor (AR) in the brain significantly mitigates PCOS symptoms (6). It has been observed that nearly 70% of PCOS patients present with high levels of LH and an elevated ratio of LH to follicle-stimulating hormone (FSH) as the pulse frequency and amplitude of LH increases. Due to the negative inhibition of estradiol, anti-müllerian hormone (AMH) is secreted by small antral follicles with a maximum diameter of 8 mm. Excess androgen increases the number of pre-antral and small follicles that are growing, resulting in a 2- to 3-fold elevation in circulating levels of AMH in PCOS patients, which further diminishes follicular maturation. High AMH is capable of directly stimulating GnRH neuron activity, favoring LH release and decreasing the sensitivity of granulosa cells to FSH (**Figure 3**). Collectively, AMH is a dual regulator of follicle growth and hypothalamic GnRH secretion, thus creating a vicious cycle (7). Moreover, hyperandrogenism disrupts the negative feedback from estradiol and progesterone on the gonadal axis. In turn, this leads to the persistent hypersecretion of LH. However, estrogen receptor α, AR, and progesterone receptors are not found in GnRH neurons. Indeed, it has been found that GnRH pulsatility is jointly controlled by upstream regulators, namely, kisspeptin, neurokinin B (NKB), and dynorphin A (DynA), also known as KNDy neurons (5). Okada and colleagues have found that hyperandrogenemia also stimulates the expression of kisspeptin and NKB, whereas it blocks the activity of DynA expression (8). Alongside this study, elevated serum kisspeptin levels in PCOS were observed; these are regarded as a major GnRH pulse generator (5). Theoretically, kisspeptin neurons are located in the anteroventral periventricular nucleus (AVPV) and arcuate nucleus (ARC) in rodents (9). In contrast, kisspeptin neurons are mainly distributed in the infundibular nucleus and the preoptic area (POA) in humans. Kisspeptin stimulates GnRH release and pulsatility by binding to its receptor (KISS1R).

## 3 Insulin resistance

Hyperandrogenism promotes the accumulation of subcutaneous fat and the development of insulin resistance by suppressing lipolysis and promoting lipogenesis of adipocytes (10). Obesity may aggravate IR: overall, it promotes insulin secretion while repressing its clearance and degradation (11). Nevertheless, IR occurs in both obese PCOS patients and those of normal weight, regardless of body mass index (BMI). Stepto and his team measured IR in different groups using the hyperinsulinemic–euglycemic clamp technique, which is regarded as the gold standard for evaluation of the beta cell response to insulin. They observed that IR was present in 75% of non-obese PCOS patients, 95% of obese patients, and only 62% of obese healthy controls (12). Hence, it can be deduced that there is an intrinsic defect in insulin receptor signaling in PCOS. Increased serine phosphorylation and reduced tyrosine phosphorylation of insulin receptors and IRS1, leading to impairment of downstream insulin signal transduction, is a primary reason for IR in PCOS (10). Interestingly, one study compared the skeletal insulin sensitivity of lean PCOS patients to their healthy counterparts, and found that there was no defect in the proximal component of insulin signaling. However, the decreased circulating adiponectin levels that impair AMPK activity, as well as the decreased response of pyruvate dehydrogenase (PDH) to insulin stimulation, were found to drive the development of IR in skeletal muscle (13). More recently, a large-scale cross-trait analysis has revealed a genetic link between type 2 diabetes and PCOS, identifying 14 related single-nucleotide polymorphisms (SNPs). Furthermore, these associations are partially independent of BMI (14). Of note, BMI assessment does not accurately reflect the distribution of fat. PCOS patients have more visceral adiposity than controls. Another study found that rs7190396, located near the FTO gene, has the most significant association with PCOS with T2D (15). One genomic study, conducted among genetically homogeneous Italian families with PCOS, identified a number of novel genes associated with PCOS risk, with more studies required for this finding to be replicated in other ethnic groups. Moreover, pathway analysis has indicated that Wnt signaling is the most-affected pathway for risk genes linked to PCOS (16). However, to date, the susceptibility loci identified by genome-wide association studies (GWAS) only explain approximately 10% of the genetic basis of PCOS (17). Overall, these genetic studies indicate that different subtypes of PCOS may share similar underlying biological mechanisms (18).

## 4 Hyperandrogenism

Patients with PCOS may experience a variety of distressing symptoms caused by excess male sex hormones, such as hirsutism (increased facial or body hair), androgenetic alopecia, and acne. The hyperactive hypothalamic–pituitary–gonadal axis in PCOS patients results in excessive ovarian androgen production in theca cells via upregulation of CYP17A1 enzyme activity (19). Additionally, hyperinsulinemia also reduces hepatic SHBG synthesis, resulting in higher free androgen levels, an active form for the elicitation of biological effects (20). It has been long assumed that ovarian-derived androgen is to blame for the metabolic abnormalities associated with PCOS. Furthermore, previous studies strongly indicate that most steroidogenic enzymes are overexpressed in the theca cells of PCOS patients (21). However, hyperandrogenism can still persist even when ovarian androgen synthesis is suppressed in PCOS patients. In recent years, it has been proposed that adrenal production of 11-oxygenated androgens is the major source of circulating male sex hormones, accounting for a greater proportion than classic androgens (22, 23). This is partly because of the over-reactivity of the adrenal zona reticularis to ACTH stimulation. These findings demonstrate that adrenal hyperandrogenism is also a cause of IR (24). Intriguingly, administration of metformin only lowers testosterone levels, while having no effect on 11-oxygenated androgens (25). Insulin induces androgen production in the adipose tissue in PCOS via upregulation of aldo-keto reductase type 1C3 (AKR1C3) activity, which can convert classical androgen and 11-oxygenated androgens into potent androgen forms, namely, T or DHT and 11-ketotestosterone(11K-T), respectively (26). Obese patients have higher 11-KT levels due to increased AT volume and AKR1C3 activity (22). Moreover, AKR1C3 activates fatty acid synthase (FASN), an enzyme for *de novo* lipid synthesis, leading to excess lipid accumulation and lipotoxicity (27). To sum up, the ovary, AT, and adrenal glands collectively participate in the hyperandrogenism and IR observed in PCOS patients. Various studies have reported that PCOS patients may face further health implications and poor metabolic status even if their BMI is normal. A correlation has been identified between the prevalence of non-alcoholic fatty liver disease (NAFLD) and hyperandrogenemia, independent of obesity (28). Moreover, women with PCOS with hyperandrogenism have a higher risk of developing T2D in later life than their counterparts with normal androgen levels (29, 30). Another study using data from the UK Biobank revealed sex differences in the effects of testosterone on certain human diseases. A genetically determined increase in testosterone by 1 s.d. increases the risks of polycystic ovary syndrome in women (OR = 1.51; 95% CI: 1.33–1.72), while lowering the risk of type 2 diabetes in men (OR = 0.86; 95% CI: 0.76–0.98) (31). These findings imply that hyperandrogenism is heritable and is not merely a major feature of PCOS; on the contrary, it might be a causative factor of this condition to some extent. Moreover, excess maternal androgen exposure would interfere with placental function and increase the risk of developing PCOS in their female offspring, as well as lowering the quality of sperm in male offspring (32, 33); specifically, this constitutes transgenerational transmission. Due to ethical considerations, animal models of PCOS have been widely established in order to better understand the etiology and pathophysiology of this disease. Dehydroepiandrosterone- (DHEA), letrozole-, and AMH-induced rodent models are frequently used for their highly PCOS-like reproductive and metabolic traits (34, 35). Prenatal androgen exposure helps us to more fully explore the mechanisms of transgenerational transmission, and gene-modified models pave the way for decisive mechanistic studies.

## 5 Inflammation and oxidative stress

It has been widely accepted that chronic low-grade inflammation is involved in the pathogenesis of PCOS, mainly owing to the excessive fat accumulation observed, particularly in the visceral adipose tissues (36, 37). Hypoxia triggers necrosis of adipose tissues, which later recruits and activates immune defense cells, initiating a complement cascade, and ultimately leading to a pro-inflammatory state. Elevated inflammatory biomarkers include high sensitivity C‐reactive protein (hsCRP), white blood cell counts, cytokines (e.g., IL‐1β, IL‐6, and IL‐18), and chemokines (e.g., MCP‐1 and MIF) (38). Mitochondrial dysfunction, obesity, IR, and unhealthy dietary patterns (for instance, high composition of dietary carbohydrates and high intake of fatty acids) are factors contributing to the development of oxidative stress (OS). OS is the result of an imbalance between oxidants and antioxidants and leads to surplus reactive oxygen species (ROS) (39). The pathological factors mentioned above, as well as hyperandrogenism (40), together play important roles in the development of low-grade inflammation in PCOS, and these factors also interact with one other, thus creating a vicious circle (41, 42). However, it is noteworthy that OS and inflammation are not always detrimental to the female reproductive system. Under some circumstances, they are necessary for ovulation and progesterone synthesis (39).

## 6 Pathogenic signaling pathways

### 6.1 The PI3K/Akt signaling pathway

The phosphoinositide-3 kinase/protein kinase B (PI3K/Akt) signaling pathway plays an essential role in cell survival and glucose homeostasis. Based on structural and functional characteristics, there are three classes of PI3K, with class 1 PI3K being the most extensively studied. When insulin is released by β cells, it binds to its receptor. Subsequently, the insulin receptor substrate (IRS) is recruited to insulin receptors, resulting in the activation and phosphorylation of PI3K. Activated class ι PI3K will then transform phosphatidylinositol 4,5-bisphosphate (PIP2) into phosphatidylinositol 3,4,5-trisphosphate (PIP3). As a second messenger, PIP3 binds to the PH domain of AKT in the plasma membrane. Furthermore, with the help of phosphoinositide dependent kinase 1 (PDK1) and mechanistic target of rapamycin kinase complex 2 (mTORC2), respectively, the threonine phosphorylation site (Thr308) and serine phosphorylation site (Ser473) on Akt are phosphorylated (43). Once activated, AKT will exert diverse biological effects downstream as a core part of the PI3K/Akt signaling pathway (44). Theoretically, FSH modulates the expression of IRS2, thus affecting glycogen synthesis and glucose uptake via the PI3K/Akt pathway (45). However, in PCOS patients, elevated LH activity interferes with the expression of FSH-stimulated IRS2. Ultimately, the defective FSH-responsiveness results in glycogen depletion and disruption to follicle growth (46). Hence, any flaw or truncation in insulin signal transduction will impair insulin sensitivity.

#### 6.1.1 The main components and regulators

In muscle or adipose tissue (AT), AKT prompts glucose transporter type 4 (GLUT4) to translocate from the cytoplasm to the cytomembrane, thereby mediating insulin-stimulated glucose uptake. GLUT4 expression in the adipocytes is observed to be reduced in PCOS, and GLUT1 gene expression is not increased in compensation (47). Interestingly, metformin medication is found to have lasting effects in PCOS patients in terms of induction of GLUT4 mRNA expression in AT, even after its withdrawal (48).

##### 6.1.1.1 Forkhead box O transcription factors

FoxOs are a group of direct downstream targets of the PI3K/Akt pathway; when AKT is activated, it will phosphorylate FoxOs and hamper their nuclear translocation, thereby inhibiting the expression of some pro-apoptotic genes. The findings of numerous studies support the view that the FOXO family plays an important role in female reproduction (39). AMH favors autophagy and inhibits activation of FOXO3A, thus preventing premature follicle depletion (49–51). Additionally, one study has demonstrated that FOXO3 expression is increased in non-obese PCOS patients and is related to m^6^A modification (52). In addition, the level of FoxO1 expression is increased in women with PCOS compared to controls, and this may be related to ovarian inflammation and follicular atresia. Previous studies have found that increased production of pro-inflammatory cytokines is positively correlated with FoxO1 expression in hepatocytes and macrophages (53, 54). Activation of FoxO1 also inhibits the gene expression of SLC2A4 (GLUT4), reducing glucose uptake and causing IR (51). Moreover, angiopoietin-like protein 2 (ANGPTL2) has been shown to be involved in IR in PCOS models via an increase in FoxO1 expression (55).

##### 6.1.1.2 Glycogen synthase kinase-3

Glycogen synthase (GS) is negatively regulated by GSK3, which inhibits glycogen synthesis; in contrast, Akt phosphorylates GSK3 to inhibit its activity. The GSK3 family consists of two isoforms in mammals: GSK3α and GSK3β. Many previous studies have reported that the GSK3β gene is over-activated in PCOS patients (56, 57). Conversely, another study has found that the phosphorylation of GSK3β at the ser9 site is increased in the uterus of rats with PCOS-like symptoms induced with a combination of insulin and human chorionic gonadotropin (hCG). In the same study, Glut7 mRNA was found to be significantly increased and a decline in Glut4 expression was observed in the endometrium in the rat model of PCOS (58).

##### 6.1.1.3 Phosphatase and tension homologue

PTEN acts as a negative regulator: it can convert PIP3 back into PIP2, thereby inhibiting activation of the PI3K/Akt pathway (43). Women with PCOS have been found to exhibit increased endometrial expression of PTEN (59). Ubiquitin-specific protease 25 (USP25) is a deubiquitinating enzyme that prevents degradation of PTEN. Notably, USP25 expression is found to be increased in PCOS patients, thus stimulating excessive PTEN synthesis (60). Serum amyloid A1 (SAA1), an acute-phase protein produced in response to inflammation, infection, and trauma, has also been found to be increased in PCOS patients. Its concentration in follicular fluid is 10 times higher than that in blood circulation. Researchers have found that this can induce the PTEN expression and, as a result, suppress downstream signaling (61).

##### 6.1.1.4 Lymphocyte adaptor protein

Many studies have confirmed that LNK (SH2B3) is a regulatory factor in insulin signaling via its effect on glucose uptake. LNK knockdown in mice results in increased GLUT4 activity in adipose tissue (62). LNK expression is significantly higher in women with PCOS with IR compared to patients without IR and control groups, and LNK suppresses activation of the insulin-meditated PI3K pathway (63). It has also been found to promote granulosa cell apoptosis via the AKT/FOXO3 pathway in PCOS patients (64).

#### 6.1.2 Therapeutic strategies

##### 6.1.2.1 Gene therapy

MicroRNAs (miRNAs) are a large family of small non-coding RNA sequences that negatively regulate gene expression post-transcriptionally (65). They are linked to many diseases, including PCOS. The follicular fluid-derived exosomal miR-18b-5p, targeting PTEN, might be considered as a potential therapy for reducing IR in PCOS (66). Additionally, miR-29c-3p improves glucose metabolism via inhibition of FOXO3 translocation (67).

##### 6.1.2.2 Traditional Chinese medicine

Several studies imply that berberine is safe and has a beneficial effect in treatment of PCOS, targeting multiple pathways, such as by decreasing the synthesis of excessive androgen, facilitating the expression of GLUT4 proteins, and alleviating IR (68, 69). It has also been found that certain Chinese decoctions, such as Heqi San, Liuwei Dihuang pills, and Guizhi Fuling Wan, have relevant physiological effects, improving insulin resistance and ameliorating sex hormone disturbances in rat models through activation of the PI3K/AKT pathway (70–72).

##### 6.1.2.3 Melatonin

MT is a neuro-hormone that is primarily synthesized and secreted by the pineal gland and is essential for circadian rhythm. As a multifunctional molecule, it also participates in the regulation of reproductive functions via multiple targets. An increasing body of literature strongly indicates that disruption to the circadian rhythm is closely linked to the progression of PCOS (73). Studies have revealed that long-term exposure to light results in increased levels of FSH and estradiol. Consistent with this, prolonged darkness has also been found to lead to hyperandrogenism in PCOS via downregulation of melatonin receptor 1A (74). The concentration of MT in follicular fluid (FF) has been found to be significantly lower in women with PCOS than in healthy controls. Interestingly, this is associated with increased levels of inflammatory markers (75). MT treatment effectively attenuates mitochondrial injury and oxidative stress in granulosa cells (GCs), functioning as a SIRT1 activator to activate the PDK1/Akt pathway (76, 77). Additionally, administration of MT may improve oocyte development by increasing the expression of oocyte maturation-related genes, namely, growth differentiation factor 9 (GDF9) and bone morphogenetic protein 15 (BMP15) (78); it also reverses the low progesterone levels observed in PCOS by upregulating StAR expression in GCs (79). MT is also capable of increasing FSH secretion via stimulation of the pituitary gland (80). Overall, MT protects ovarian functions by mitigating inflammation, cellular apoptosis, and OS; these effects are primarily or partially dependent on activation of the PI3K/AKT pathway.

### 6.2 The TLR4/NFκB signaling pathway

Toll-like receptor 4 (TLR4)/nuclear factor-κB (NF-κB) signaling has been extensively studied and is a well-established signaling pathway mediating inflammatory responses. Working as a pattern recognition receptor (PRR), TLR4, unlike other members of the toll-like receptor (TLR) family, can engage all four kinds of toll-interleukin receptor (TIR) domain-containing adaptor proteins: MyD88, TIRAP, TRAM, and TRIF (81). In general, with the assistance of co-receptors CD14 and MD2, TLR4 is activated by pathogen-associated molecular patterns (PAMPs), such as LPS, and damage-associated molecular patterns (DAMPs), e.g., oxLDL and saturated fatty acid; TIRAP-MyD88 adaptors are subsequently recruited to TIR domains. Eventually, the downstream IKK complex is activated, causing the IκB protein to be degraded; NF-κB subsequently enters the nucleus, further initiating the expression of a series of inflammatory molecules, such as TNF-α, IL-1, and IL-18 (82, 83). One study has indicated that over-expression of MIF stimulates hyperactivity in the NF-κ pathway in a DHEA-induced PCOS model, whereas the application of MIF antibody is associated with significant recovery from PCOS symptoms (38). Hu et al. have demonstrated that endometrial inflammation in PCOS patients is induced by TLR4/IRF-7/NFκB signaling (84). High levels of TNF-α may repress endometrial GLUT-4 expression in PCOS via NFκB activation, resulting in endometrium dysfunction (85, 86). Additionally, elevated inflammatory factors are observed in both obese patients and those of normal weight, with higher levels in the obese group (87), suggesting that obesity may exacerbate inflammatory states (88). This chronic inflammation is thought to be systemic rather than regional, with inflammatory markers travelling through the bloodstream to the ovaries, altering the micro-environment of the follicular fluid, thereby triggering the inflammatory cascade and leading to dysfunctional and aberrant GCs (89). Furthermore, miR-93-5p has also been found to contribute to the progression of apoptosis and ferroptosis in GCs in PCOS via NF-κB signaling (90).

#### 6.2.1 Inflammation and insulin resistance

High-mobility group box 1 (HMGB1) is a multi-functional small nuclear protein regulating gene transcription (91). It functions as a DAMP for activation of TLR4 signaling, with the help of MD-2, when it is released into the extracellular matrix (92). HMGB1 is required in order for LPS and nucleic acids to manifest their full toxicity (93). Circulating HMGB1 has been found to be increased in PCOS. HMGB1 has been linked to inflammation, impaired insulin sensitivity, and endothelial dysfunction in PCOS patients (94, 95). At the transcriptional level, HMGB1 is also the direct mRNA target of miR-129 (96), while lncRNA ZFAS1 could competitively bind to miR-129 to induce HMGB1 expression. Moreover, the abundance of miR-155 in the FF in PCOS is negatively correlated with HMGB1 concentration (97). The enhanced autophagy induced by increased HMGB1 content may be a contributing factor in IR in the GCs of PCOS patients, embodied by over-expression of ATG7 and a decrease in p62 levels (98). The release of HMGB1 must undergo acetylation and nuclear-to-cytoplasmic translocation processes (99). Thus, increasing the activity of Sirtuin1 (SIRT1), an NAD^+^ dependent deacetylase, can prevent HMGB1 from being released into extracellular space, thereby reducing the inflammatory response (100). Hence, the alleviation of the metabolic disorders seen in PCOS by treatment regimens such as melatonin, resveratrol, and metformin is at least partially mediated by the activation of SIRT1 (77, 101, 102). After treatment with myo-inositol (MYO) in combination with alpha-lipoic acid (ALA), levels of HMGB1 in adolescents with PCOS have been found to return to normal (25, 103). Cryptotanshinone (CRY) also exerts its therapeutic efficacy via HMGB1/TLR4/NF-κB signaling (104).

#### 6.2.2 The inflammasome and exosomes

The inflammatory cytokines L-1β and IL-18 are found to be increased in PCOS patients; these are related to ovulation, and moreover, the structure of IL‐18 is similar to that of the IL‐1 family. Maturation of these two inflammatory cytokines occurs by means of the activation of inflammasomes (105). Inflammasomes are multiple-protein complexes that are assembled and initiate inflammation in response to harmful stimuli. Dysregulation of inflammasomes is closely linked to numerous human diseases, including neurodegenerative diseases, cancers, and vascular diseases. There are two categories of inflammasomes: canonical and non-canonical inflammasomes. Generally speaking, the canonical inflammasomes are comprised of three components: sensor proteins (such as NLRP1, NLRP3, NLRP6, NAIP/NLRC4, AIM2, and PYRIN), the adaptor ASC, and effectors (pro-caspase1). When DAMPs or PAMPs are recognized by the PRRs, ASC and pro-caspase-1 are recruited to the complex. Subsequently, pro-caspase-1 is activated to form caspase-1, and pro–IL-1β, pro–IL-18, and gasdermin D are then cleaved into active forms (106). Among these, NLRP3 and AIM2 inflammasomes have been detected in PCOS patients; their presence may be driven by hyperandrogenism and fatty acids (107–109). Acetate, a histone deacetylase inhibitor, can suppress the activation of the NLRP3 inflammasome and restore the overactive kisspeptin system in rat models (110). MiR-1224-5p may inhibit activation of the NLRP3 inflammasome by targeting FOXO1 (111). Pioglitazone and metformin dual therapy could mitigate the psychological distress of PCOS patients by reducing NLRP3 inflammasome activation (112, 113), while plumbagin has been found to reduce pyroptosis of GCs. The latter inhibits the activity of WTAP, a key regulator of the RNA N6-methylase complex, consequently destabilizing the ASC mRNA and inhibiting activation of the NLRP3 inflammasome (114).

Exosomes are cell-created extracellular vesicles with sizes in the range of 50–150nm, containing various bioactive molecules. They can be transferred from one cell to another, thus enabling signal transduction and intercellular communication. In recent years, researchers have identified the dual regulatory role played by exosomes, which can negatively or positively affect the activation of inflammasomes. The function of an exosome is dependent on which cargos it carries and where it originates (115). The S100‐A9 protein in exosomes from the follicle fluids of PCOS patients may activate the NF‐κB signaling pathway (116), while human umbilical cord mesenchymal-stem-cell-derived exosomes (hUC-MSC-exos) can inhibit NF-κB signaling via stimulation of the phosphorylation and degradation of Ik-B, increasing the expression of anti-inflammatory cytokines IL-10, and are capable of activating M2 macrophage polarization. As a consequence, inflammation in the ovary is ameliorated (117). Exosomal miR-323-3p derived from adipose mesenchymal stem cells (AMSCs) can protect cumulus cells from apoptosis via direct inhibition of the over-expression of PDCD4 (PMID: 31549864). Moreover, it has been reported that the miR-21-5p carried by AMSC-EXOs can migrate to the liver and activate the IRS1/AKT pathway, thereby mitigating hepatic IR (118). To summarize, these findings indicate that exosomes derived from stem cells can inhibit inflammation through a variety of mechanisms, with microRNAs in stem cells serving as the key players (119, 120). Additionally, hsa-miR-1299, hsa-miR-6818-5p, hsa-miR-192-5p, and hsa-miR-145-5p are highly expressed in the blood of PCOS patients; therefore, these exosomal miRNAs might be promising biomarkers for the early diagnosis of PCOS (121, 122).

#### 6.2.3 The gut microbiota and inflammation

Accumulating evidence has demonstrated the close relationship between the gut microbiome and PCOS. Various studies have indicated that the composition of the gut microbiota is altered in PCOS patients, with reduced alpha and beta diversity and decreased Lactobacilli, Bifidobacteria, and Prevotellaceae content, while the abundance of Bacteroides vulgatus is increased (123, 124). This disturbance of the intestinal flora disrupts the integrity of the gut barrier, increasing gut permeability to LPS, and the resultant activation of the TLR-4/NF-κB-mediated inflammatory response. Moreover, these changes are also influenced by HA. It has been proposed that prenatal androgen exposure could lead to dysbiosis of the gut microbiota. One study has revealed an inverse correlation between alpha diversity and circulating testosterone levels (125).

#### 6.2.4 Therapeutic strategies

Supplementation with α-Linolenic acid extracted from flaxseed oil has exhibited strong potential in mitigating PCOS-related symptoms: it significantly reduces the levels of LPS and inflammatory factors, alters microbiota composition, and increases the abundance of short-chain fatty acids (125). Additionally, it has been indicated that regimens such as the Bu Shen Hua Zhuo formula and the Shaoyao-Gancao decoction relieve the symptoms of rats with PCOS by modifying the gut microbiota to suppress inflammation (126, 127). Furthermore, IL-22 links intestinal bacteria to IR and androgen excess. Serum IL-22 concentrations are significantly lower in PCOS individuals. In terms of mechanism, increased levels of Bacteroides vulgatus decrease the levels of conjugated bile acids. This results in downregulation of intestinal type 3 innate lymphoid cells (ILC3s) to produce IL-22 in a GATA3-dependent manner (128). However, treatment to increase IL-22 inhibits the release of inflammatory cytokines CCL2, CCL20, and IL-1β, ultimately improving ovarian function (124).

##### 6.2.4.1 Nutritional supplements

Some edible supplements, including coenzyme Q10, fish oil, omega-3 fatty acids, and vitamin E, have shown effectiveness in suppressing the gene expression of some inflammatory mediators, including IL-1, IL-8, and TNF-α, in women with PCOS (129–131).

### 6.3 The Keap1/Nrf2 signaling pathway

The Kelch-like ECH-associated protein 1 (Keap1)/nuclear factor erythroid 2-related factor 2 (Nrf2) system functions as a primary antioxidative defense in humans. Keap1 works as a sensor for ROS or electrophiles, whereas Nrf2 acts as a transcriptional factor that regulates the bulk of antioxidant gene expression. Under normal conditions, Nrf2 binds to the KEAP1-RBX1-CUL3 E3 ubiquitin ligase complex, and is then degraded by the ubiquitin–proteasome system in the cytoplasm. Additionally, the activity of Nrf2 is also regulated by p62, a substrate of autophagy, via the inactivation of Keap1 (132). However, in a stressed state, the structure of Keap1 changes, thus preventing Nrf2 degradation. Subsequently, the newly synthesized Nrf2 translocates to the nucleus, where it heterodimerizes with sMAF proteins. The heterodimer then recognizes AREs/EpREs, leading to the activation of downstream Nrf2-mediated genes, promoting the synthesis of certain antioxidant proteins or detoxification enzymes, such as heme oxygenase-1 (HO-1) and NADPH quinone oxidoreductase-1 (NQO-1). HO-1 is a member of the heme oxygenase family, encoded by the HMOX1 gene. As a rate-limiting enzyme, HO-1 catalyzes heme into biliverdin, carbon monoxide (CO), and free iron (Fe^2+^); biliverdin can then be converted into bilirubin. These metabolic molecules are thought to be potent antioxidants. Serum levels of HO-1 are considerably lower in non-obese PCOS patients, due to its exhaustion (78). One study has demonstrated that the miR-873-5p inhibitor can reduce ROS by directly targeting HO-1 (133). Melatonin supplementation also improves the poor quality of oocytes by suppressing nitric oxide (NO) synthetase and NO release, upregulating Nrf2 and downstream HO-1 (78). Humanin is a mitochondria-derived peptide. It has been reported that supplementing with humanin analogue (exogenous humanin) improves both IR and OS in PCOS patients via the Keap1/Nrf2 and PI3K/Akt pathways (134, 135).

#### 6.3.1 Crosstalk between NF-κB and the Keap1/Nrf2 pathway

**Figure 4:**
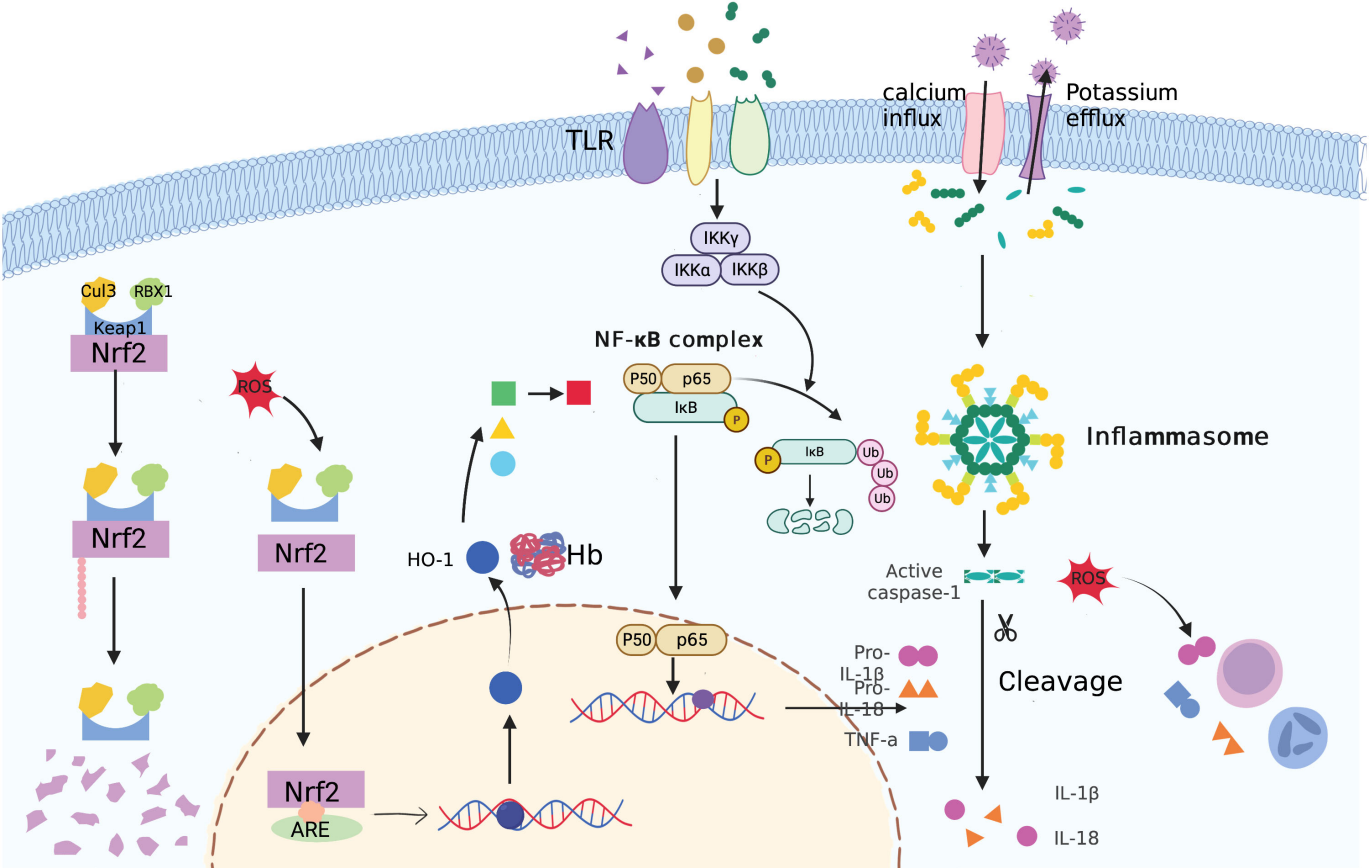
An illustration of inflammasome, TLR4/NFκB, and Keap1/Nrf2 signaling pathways in PCOS pathogenesis.

Many previous studies have demonstrated the complex interactions between the keap1/Nrf2 and NF-κB pathways in anti-inflammatory and proinflammatory responses (136). First, Nrf2 activation may inhibit the release of certain inflammatory cytokines, cell adhesion molecules, and matrix metalloproteinases (MMPs). As a result, NF-κB activation is suppressed. Additionally, Nrf2 may disrupt the NF-κB nuclear translocation process (137). On the transcriptional level, NF-κB competes with Nrf2 for the binding site of CBP, a co-activator of Nrf2. Additionally, p65 may recruit the co-repressor HDAC3, decreasing the expression of downstream ARE-driven genes. Moreover, Nrf2 may interfere with the assembly of the NLRP3 inflammasome. Therefore, Nrf2 inducers have strong potential for use in anti-inflammatory therapeutic strategies in PCOS (**Figure 4**).

#### 6.3.2 Crosstalk between AMPK and the Keap1/Nrf2 pathway

It has been demonstrated that, as a upstream modulator, AMPK activates NRF2 by promoting Nrf2 phosphorylation at serines 374, 408, and 433 (138). However, the interesting aspect of this is that, when AMPK kinase is inhibited by compound C, the basal level of Nrf2 is unaffected (139). AMPK may also regulate Nrf2 activity via an indirect pathway: it may deactivate GSK3β via PTEN–PI3K–AKT–GSK-3β cascade, thereby promoting Nrf2 ubiquitylation and degradation. Sulforaphane (SFN), considered to be a potent Nrf2 inducer, has extensive presence in certain cruciferous vegetables, including broccoli and kale (140). Numerous studies have proven its anti-aging, anticancer, and antioxidant properties via various mechanisms (141, 142). In the ovary, SFN reduces tumor invasion and angiogenesis through inhibition of the HIF-1 pathway (143). SFN has also been shown to exert a protective effect on GCs in PCOS patients via activation of the AMPK/Nrf2 pathway (144). Additionally, Ji and colleagues found that salidroside may reduce ROS production and apoptosis of GCs in PCOS patients through activation of AMPK/Nrf2 signaling (145).

#### 6.3.3 Nicotinamide adenine dinucleotide phosphate oxidase 4

NOX4 belongs to the nicotinamide adenine dinucleotide phosphate (NADPH) oxidase (NOX) family, which is a primary participant in endogenous ROS production (146). One publication has reported the involvement of NOX4 in inducing ROS production and impairing skeletal insulin sensitivity in testosterone-induced models of PCOS (147). Researchers have also demonstrated that NOX and Keap1/Nrf2 play interacting roles in the control of ROS levels. NOX4 deficiency has been shown to ease the symptoms of rats with PCOS (148). Intriguingly, over-expression of NOX4 may in turn activate expression of Nrf2, thus exerting a protective effect on cardiomyocytes under chronic conditions of overload (149). However, similar phenomena have not been observed in the case of lung or kidney injuries (150).

### 6.4 The AMPK pathway

Adenosine monophosphate (AMP)-activated protein kinase (AMPK), which is present in almost all eukaryotes, is a highly conserved heterotrimeric serine/threonine protein kinase that regulates energy metabolism (151). It consists of α, β, and γ subunits, among which α has α1 and α2 subunits, β has β1 and β2 subunits, and γ has γ1, γ2, and γ3 subunits. When there is an energy deficiency, the ratio of AMP to ATP rises and AMPK is activated to produce more or consume less ATP in order to maintain energy balance (152). Reduced expression of α1AMPK is observed in the GCs of women with PCOS, and this is accompanied by higher steroidogenesis due to the increased activity of 3β-hydroxysteroid dehydrogenase (3βHSD) and P450 side-chain cleavage enzyme (P450scc). With the deletion of α1AMPK in rodent models, antral follicle numbers are consistently higher, along with a reduction in the population of pre-ovulatory follicles (153). Glycolysis is found to be enhanced in the GCs of PCOS patients. This increased activity of glycolysis is a marker of activated mTOR signaling and inactivation of AMPK, resulting in the excessive activation of primordial follicles and a reduction in resting follicle storage (154). Another study has found that the skeletal IR of PCOS patients of normal weight is not related to proximal defects of the insulin signaling pathway, but is instead associated with circulating adiponectin levels and subsequent inhibition of AMPK kinase (13, 155). Of note, more recent research has found that AMPK activity is suppressed in subcutaneous adipose tissue (SAT), while insulin sensitivity is preserved in visceral adipose tissue (VAT) by upregulation of AMPK via overfeeding in rat models of PCOS (156). Hence, the theory of limited expandability of SAT has been proposed: namely, that ectopic fat deposition in the muscle and liver and IR are followed by excessive fat accumulation in SAT (157).

#### 6.4.1 Crosstalk between AMPK and SIRTs

SIRTs are a family of NAD^+^-dependent histone deacetylases that regulate a variety of physiological functions. To date, seven members of the family (SIRT1–SIRT7) have been identified. SIRT1 can deacetylate LKB1, an upstream regulator of AMPK, increasing the cytoplasmic localization of LKB1 and thus promoting AMPK activation. In turn, AMPK enhances SIRT1 activity by boosting cellular NAD+ levels. Loss of SIRT1 leads to a larger population of early-stage follicles and to ovulation (158). A number of reports have demonstrated that increasing SIRT1 activity can alleviate PCOS (159). Additionally, SIRT1 helps with scavenging of advanced glycation end products (AGEs) through upregulation of glyoxalases, a key component of the anti-glycation defense system (160). As a mitochondrial protein, SIRT3 is found to be decreased in PCOS, and this deficiency contributes to the over-production of ROS and decreased mitochondrial membrane potential (161). Proliferator-activated receptor gamma coactivator-1alpha (PGC-1alpha) acts on the murine SIRT3 promoter; the resulting increase in expression of SIRT3 protects the mitochondria from OS damage (162). Interestingly, SIRT3 levels are also elevated in DHT-induced models of PCOS, supporting the concept of adaptive response (160). The increased expression of SIRT1 and SIRT3 induced by low-level oxidative stress may be the result of a compensatory defense, thus favoring autophagy, which is characterized by increased levels of autophagy marker LC3-II and decreased P62 levels in GCs; consequently, the effect is to promote ovarian health. Studies have shown that androgen can induce autophagy in the GCs in PCOS in a dose-dependent pattern; elevated serum homocysteinem levels are also involved (163, 164). Notably, a harsh environment might even lead to degradation of proteasome-mediated SIRTs and inhibition of autophagy.

#### 6.4.2 Therapeutic strategies

In theory, restoration of AMPK expression is expected to be a potential therapeutic strategy.

#### 6.4.2.1 Drugs

Metformin is a well-known AMPK activator: it phosphorylates AMPK at the T172 site, thus blocking the expression of matrix metalloproteinase-2 (MMP2) and MMP-9, which may be related to abnormal follicular atresia (165, 166). A glucagon-like peptide 1 (GLP-1) receptor agonist can also be prescribed in order to lower glucose levels. Exenatide and liraglutide have both exhibited beneficial effects in PCOS patients when used alone or in combination with metformin, working via the AMPK/SIRT1 pathway (102, 167). Myo-inositol (MYO) plays a role in improving insulin sensitivity by activating AMPK and increasing GLUT-4 levels. Previous clinical trials have demonstrated that MYO treatment improves outcomes for PCOS patients who are receiving treatment with assisted reproductive technology (168, 169). Moreover, calcitriol supplementation elicits a cardioprotective effect by blunting PCOS-related cardiac remodeling (170). Considering the decreased NAD+ levels occurring in the GCs of PCOS patients, nicotinamide (NAM) and its metabolite N1-Methylnicotinamide (MNAM) have been shown to have therapeutic potential in PCOS (171, 172). In terms of the mechanism, low-dose MNAM supplementation triggers transient ROS generation induced by increased aldehyde oxidase 1 (AOX1) activity. In addition, these agents alleviate hyperandrogenism through inhibition of AMPK-activation-mediated expression of CYP17A1 (173).

#### 6.4.2.2 Phytochemicals

Quercetin (QUR), a natural phytochemical with a high flavonol content, can help to ameliorate metabolic and endocrine abnormalities in PCOS, such as by improving IR, inflammation, and hyperandrogenism (174). Upon treatment with QUR, activity of AMPK and SIRT1 is increased, reducing fat accumulation by modulating the secretion of adipocytokines (158, 175). Other compounds, including fisetin and baicalin, have both exhibited beneficial effects via targeting of AMPK (176, 177).

### 6.5 The MAPK pathway

The mitogen-activated protein kinase (MAPK) signaling cascades are composed of three main kinases: MAPK kinase kinase kinase (MAPKKK), MAPK kinase kinase (MAPKK), and MAPK. These transmit external signals to downstream effectors step by step to regulate a number of critical biological processes (178). Three classic MAPK signaling pathways are extracellular signal-regulated kinase (ERK), p38 MAPK, and c-Jun N-terminal kinase (JNK). ERK is mainly activated by stimuli such as growth factors and mitogens, whereas JNK and p38 are often activated by stress and inflammatory mediators (179). One study has identified a possible role of IL-15 in the development of PCOS: the increased IL-15 level is derived from GCs and induces the phosphorylation of p38 MAPK and JNK, promoting excessive androgen synthesis (180). It has been verified that plasma leptin may be a reliable predictor of PCOS, and high leptin levels are strongly associated with both hyperandrogenism and hyperinsulinemia (181). Additionally, high leptin concentrations may cause oocyte dysfunction (182). Decreased expression of Sam68, an RNA binding protein, links the elevated serum leptin levels to HA. Mechanically, downregulation of Sam68 blocks the aromatase enzyme in response to leptin, thus causing leptin resistance (183), while over-expression of Sam68 restores insulin sensitivity by modulating IRS-1 expression to activate the PI3K and MAPK pathways (184). These results illustrate the dual regulatory functions fulfilled by Sam68 in endocrine dysfunction among PCOS patients. Increased p62 levels in the theca cells in PCOS, representing inhibition of autophagy, cause oxidative damage and hyperandrogenism via activation of the p38 and JNK pathways (185). It is of interest that autophagy is enhanced in GCs and causes GC apoptosis. Under DHT stimulation, the promoters of Map3k1 and Map1lc3a are hypomethylated, leading to increased expression of autophagic-related proteins. This finding implies that MAPK/p53 pathway activation of GCs is driven by dihydrotestosterone (DHT) at the transcriptional level (186). As a result of its binding to the EGFR, elevated levels of heparin-binding epidermal growth factor-like growth factor (HB-EGF) in the FF of PCOS patients activate the cAMP-PKA-dependent JNK and ERK1/2 pathways. Consequently, FOXO1 promotes Ca2+ influx and initiates excessive estrogen synthesis, ultimately leading to estrogen-induced mitochondrial dysfunction and apoptosis of GCs (187). However, other studies have revealed inhibition of the ERK pathway and reduced HB-EGF levels in PCOS patients (188). Expression of StAR and subsequently increased progesterone synthesis could be modulated by upregulation of ERK activity (189, 190). A series of studies have demonstrated that administration of an NK3R antagonist (fezolinetant) leads to a reduction in LH secretion and attenuates symptoms of PCOS by reducing GnRH pulse generation (191). Of note, a recent study has indicated that NKB and NK3R genes are also highly expressed in the ovary, over-activating the MAPK-ERK pathway and disrupting oxidative metabolism (192, 193).

#### 6.5.1 Therapeutic strategies

##### 6.5.1.1 Drugs

The medication metformin has also been found to alleviate ER triggered by hyperandrogenism via repressing the p38MAPK signaling (194). It has been reported that a 400 mg/kg dose of berberine significantly improves insulin sensitivity in rats by increasing GLUT4 expression via inactivation of MAPK and activation of PI3K/AKT signaling (69).

Cangfudaotan decoction exerts an anti-apoptotic effect and restores mitochondrial functions via modulation of the phosphorylation of apoptosis signal-regulating kinase 1 (ASK1) protein; this suppresses downstream JNK activation, thus upregulating Bcl-2 and Bax and cleaving caspase-9 and -3 to limit apoptosis (195).

##### 6.5.1.2 Brown adipose tissue transplantation

BAT activity is dysregulated in PCOS, with an increase in white adipose tissue in its place. Excess androgens inhibit the thermogenesis of BAT and mitochondrial biogenesis, with a reduction in the thermogenic markers uncoupling protein type 1 (UCP1) and proliferator-activated receptor gamma coactivator-1 alpha (PCG-1) (196). However, BAT transplantation from healthy recipients with ample bioactive compounds, such as adipocytokine, could restore BAT function. KEGG pathway analysis has revealed that the MAPK, PI3K and Wnt pathways are involved in this process (197, 198).

##### 6.5.1.3 Gene therapy

miR-14 has been found to exert a protective role in GCs in PCOS patients via inhibition of p38 MAPK and ERK signaling (199).

### 6.6 The JAK/STAT pathway

The Janus kinase (JAK)/signal transducer and activator of transcription (STAT) pathway is crucial for cell proliferation, differentiation, and apoptosis. The JAK family consists of four members, namely, JAK1, JAK2, JAK3, and TYK2, and there are seven types of STATs (200).

When a cytokine binds to its corresponding receptor, the receptor-associated JAK is activated. This then stimulates the phosphorylation and dimerization of the STAT. Subsequently, the activated STAT dimer translocates to the nucleus, where it regulates the expression of target genes (201). In the bovine ovary, STAT4 is regarded as a transcription regulator in follicular growth (202). A polygenic integrative analysis has provided evidence that STAT function is significantly dysregulated in the ovary in PCOS (203). It has also been confirmed that the levels of phosphorylated STAT3 are elevated in the placentas of PCOS patients (204). The elevated IL-6 levels observed in PCOS, particularly in the obese phenotype, might be a possible explanation. Over-expression of IL-6 and IL-11 stimulates the proliferation of adipocytes in rat adipose tissues, which is followed by activation of the STAT3 pathway (205). In trans IL-6 activation, the highly expressed IL-6 firstly binds to its soluble receptor (sIL-6R); the complex later binds to gp130 molecules, thus mediating downstream JAK/STAT3 activation and exerting its pro-inflammatory nature in PCOS patients. The classical signaling pathway, in contrast, is anti-inflammatory and is initiated by IL-6 binding to membrane-bound IL-6R with the aid of gp130 (206).

#### 6.6.1 Therapeutic strategies

**Figure 5:**
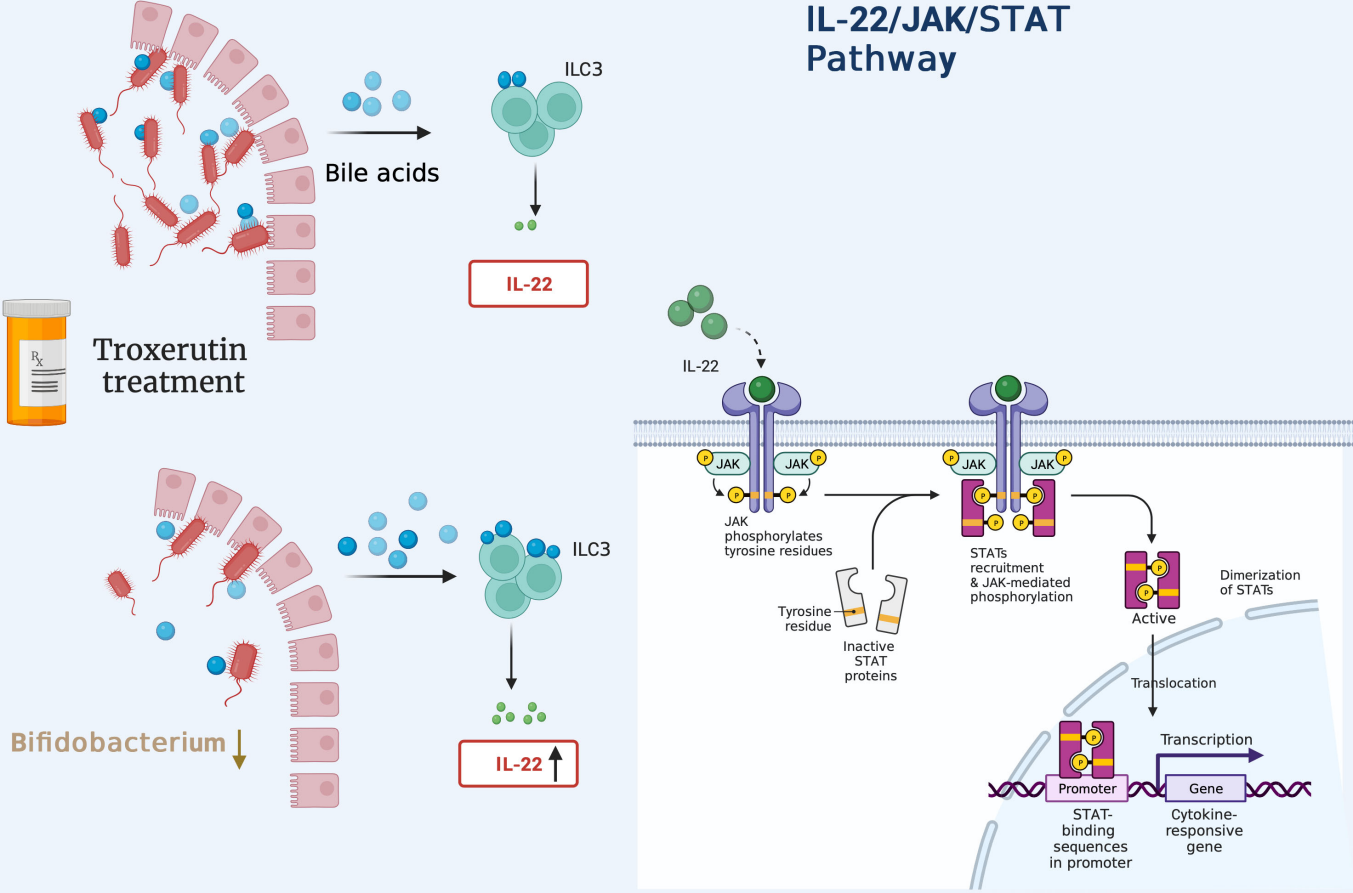
Administration of troxerutin decreases the abundance of Bifidobacterium, inducing the production of more unconjugated secondary bile acids. These bind to TGR5 receptors present in ILC3 cells to secrete more IL-22 in a GATA-dependent manner, thus activating downstream JAK1/STAT3 signaling.

miR-520h has been found to directly target IL-6R, thereby inhibiting the development of PCOS (207). Tofacitinib, one type of JAK inhibitor, was initially developed for the treatment of autoimmune diseases, such as rheumatoid arthritis (RA) (208). It is of interest that inhibition of miR-199a-5p reduces apoptosis in GCs of PCOS patients via activation of JAK/STAT3 pathway. However, this protective effect is counteracted by treatment with tofacitinib (209). Moreover, the flavonoids extracted from Nervilia fordii have shown potent effectiveness in reversing PCOS-related traits via regulation of IL-6 through the JAK2/STAT3 pathway (210). A troxerutin regimen is reported to decrease IR through activation of JAK1/STAT3 signaling in beta cells (211); this decreases the abundance of Bifidobacterium and alters the bile acid profile, ultimately boosting serum IL-22 levels (**Figure 5**).

### 6.7 The Hippo pathway

**Figure 6:**
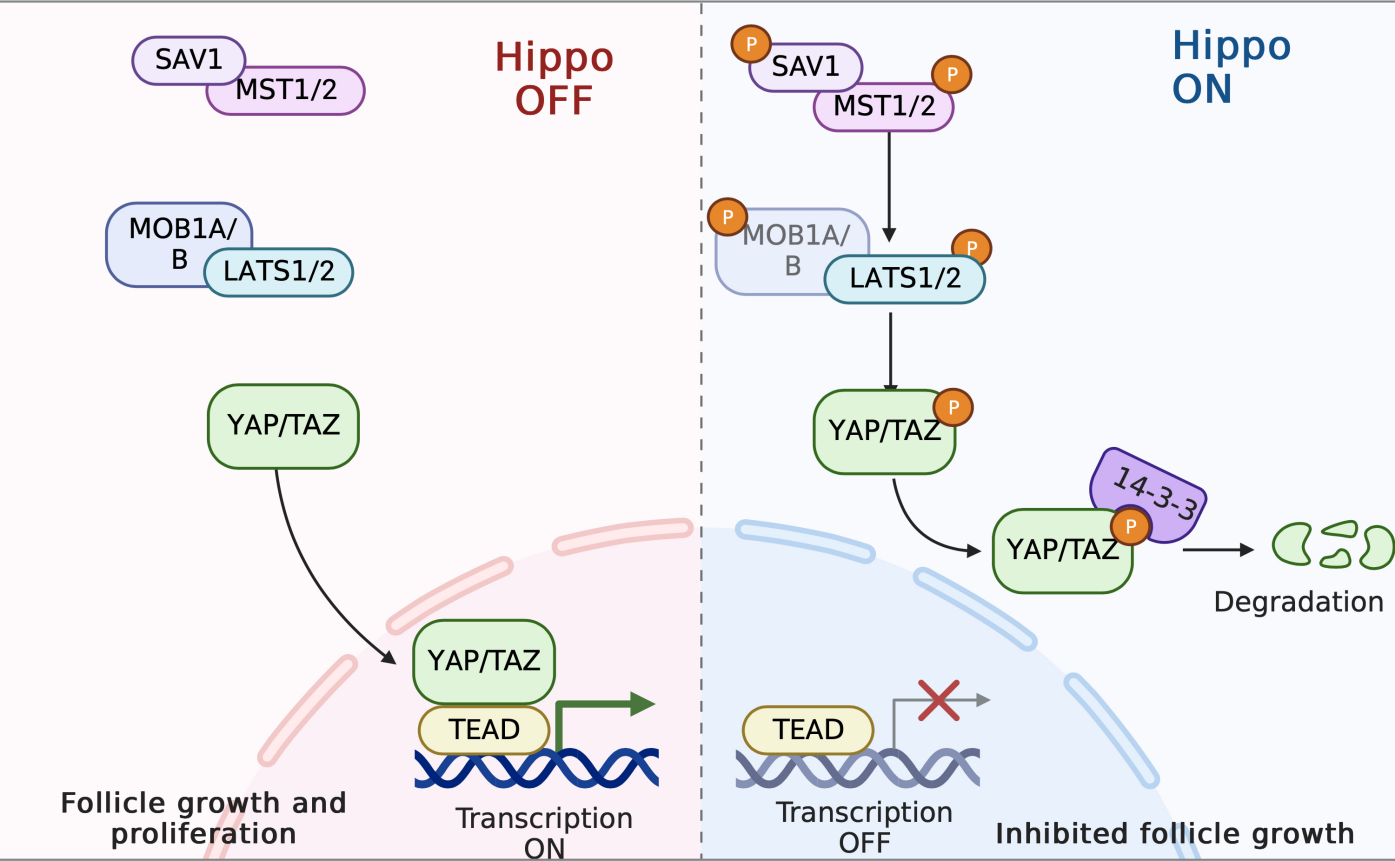
The Hippo pathway in PCOS pathogenesis. When the Hippo pathway is active, the MST1/2–SAV1 complex phosphorylates and activates the LATS1/2–MOB1A/B complex; this then directly phosphorylates YAP and TAZ. Consequently, YAP/TAZ is sequestrated in the cytoplasm by 14-3-3 proteins and subsequently degraded. Follicle growth is inhibited. In contrast, disruption of Hippo signaling leads to nuclear translocation of YAP and increased downstream CCN growth factor transcription, thereby stimulating follicle growth.

The Hippo pathway, which was originally discovered in Drosophila, is a highly conserved signaling cascade that plays significant biological roles in controlling organ size, tissue homeostasis, and regeneration (212). The Hippo pathway, unlike other ligand-induced signaling pathways, lacks ligands or receptors (213). The mammalian Hippo pathway cascade is made up of core kinases—mammalian Ste20-like protein kinase ½ (MST1/2) and large tumor suppressor ½ (LATS1/2)—as well as adaptor proteins [Salvador (SAV1) and Mps one binder 1A/B (MOB1A/B)] and the downstream transcriptional co-activators yes-associated protein (YAP) and transcriptional co-activator with PDZ-binding motif (TAZ). The Hippo pathway is modulated by a number of upstream signals, such as mechanical force, G protein-coupled receptors (GPCRs), cell polarity, and cell–cell contact. When the Hippo pathway is active, the MST1/2–SAV1 complex phosphorylates and activates the LATS1/2–MOB1A/B complex, which then directly phosphorylates YAP and TAZ. YAP/TAZ is sequestered in the cytoplasm through 14-3-3 protein binding and subsequently degraded by the proteasome (214). Conversely, when the Hippo pathway is inactive, YAP and TAZ are dephosphorylated and translocate to the nucleus, where they bind with transcription factors (TEADs) to increase the expression of downstream targets: growth factors (CCN2,3) and apoptosis inhibitors (BIRC1,7) (215). These proteins, in turn, stimulate ovarian cell growth and proliferation (216) (**Figure 6**). Emerging studies have demonstrated the involvement of the Hippo signaling pathway in the regulation of all stages of follicle development (217). The rigid environment of the ovarian cortex activates the Hippo pathway, inhibiting the follicles from entry into the growth phase, thus maintaining the primordial follicles in a dormant state. As follicles grow, they move to the medullar region, which can offer a softer matrix for proliferation and expansion (218). Of note, the modulation of local Hippo signaling in the follicles varies: for example, larger follicles may disrupt the growth of neighboring smaller follicles by enhancing Hippo signaling, resulting in the maturation of a single oocyte. In mice, as the ovary ages, the levels of MST1 and LATS2 declines, while a similar linear correlation is not observed in the case of YAP levels. In addition, premature ovarian failure during later life has observed in LATS1/2-deficient mice.

It has been demonstrated that disruption of the Hippo signaling pathway is associated with PCOS, premature ovarian failure (POI), and ovarian tumor (219, 220). GWAS have revealed that SNP rs11225161 and rs11225138 from the YAP1 gene are associated with PCOS, specifically with impaired glucose tolerance and high LH levels, respectively (221). Another two loci, rs113168128 (ERBB4) and rs144248326 (WWTR1), have recently been identified and linked to anovulation (222). Of note, similar associations between YAP1 and the SNPs mentioned above have not been detected in teenagers with PCOS (223). Furthermore, one study has found that the YAP1 promoter is hypo-methylated in the GCs of PCOS patients, thus resulting in increased YAP1 mRNA and protein levels. Additionally, this study revealed a negative correlation between the degree of methylation of the YAP1 promoter and androgen levels, in a dose-dependent manner (224). Mechanically, given the sensitivity of Hippo signaling to physical force, the effect of the fibrotic extracellular matrix (ECM) and the thickened and sclerosed cortex of the polycystic ovaries, which contains more F-actin and collagen, is to downregulate Hippo signaling, resulting in YAP1 over-activation, and thus leading to stromal hypertrophy and over-proliferation of theca cells. Finally, this stimulates hyperplastic theca cells to over-produce androgen and causes multiple small immature follicles to be arrested simultaneously (213). Notably, inhibitory YAP1 activity mediated by PKA is required for an LH surge to trigger ovulation (225, 226). In reaction to ovulatory signals, amphiregulin (Areg) initiates transcription under LH pulse stimulation, which triggers the subsequent resumption of meiosis and ovulation. However, Areg concentrations are lower in PCOS, with unchanged LH receptor levels. In addition, it has been revealed that Areg promoter is a direct target of YAP1 (227, 228). Taken together, these findings indicate that the activation of YAP1 is essential for GC proliferation prior to ovulation, but its persistent over-activation may cause anovulation and infertility through interference with AREG expression.

#### 6.7.1 Interplay with AMPK

Compelling evidence has revealed that YAP1/TAZ activity can be modulated by metabolism (229). AMPK is a key energy sensor; it negatively regulates YAP1 activity by activating LATS1/2 through AMOTL1 or by directly phosphorylating YAP1 at Ser 94. Subsequently, YAP1 is sequestered in the cytoplasm and inhibits downstream gene transcription. In concert with prior studies, metformin, a well-known AMPK activator, has been reported to target YAP1 at S127 (230). Findings also indicate that organ or tissue size is precisely controlled and coordinated with energy status. When there is limited energy availability, the Hippo pathway is activated to support basic survival rather than cellular proliferation (231). Under conditions of sufficient nutrients, however, the high level of YAP activity enhances glycolysis and activates mTOR, leading to excessive activation of dormant primordial follicles (154).

#### 6.7.2 Therapeutic strategies

Wedge resection and laparoscopic ovarian drilling (LOD) have been successfully used to treat PCOS by normalizing ovulation (229). It has been found that LOD may loosen cortical layers via alteration of ovarian mechanical forces and destruction of hyperplastic stromal tissue through thermal effects (232), thus decreasing androgen synthesis and normalizing the HPO axis. *In vitro* activation (IVA) with the administration of actin polymerization—μM jasplakinolide (JASP) or sphingosine-1-phosphate (S1P)—could be a novel therapy for PCOS patients with fewer operative complications. This can convert G-actin into F-actin, increasing YAP nuclear translocation and thereby causing the disrupted follicular growth in PCOS patients to be resumed.

### 6.8 The Wnt pathway

The Wnt signaling pathway, which is evolutionarily conserved, is also essential for cell proliferation, differentiation, apoptosis, and tissue homeostasis. Dysregulation of this pathway is associated with many types of developmental disorders, fibrosis, and oncogenesis (233). In mammals, there are 19 Wnt ligands, 10 Frizzled (FZD) receptors, and 3 Dishevelled (DVL) proteins. Wnt signaling can be subdivided into two types: β-catenin-dependent signaling (the canonical pathway) and β-catenin-independent signaling (the non-canonical pathway), with the latter further divided into the Wnt/planar cell polarity (PCP) and Wnt/calcium(Ca^2+^) pathways (234).

#### 6.8.1 Wnt/β-catenin signaling

In the case of Wnt/β-catenin signaling (the canonical pathway), when the ligands (such as Wnt1, Wnt2, Wnt3, Wnt8a, and Wnt10b) bind to the Frizzled (FZD) receptors and LRP5/6 co-receptors, signaling is activated, leading to the recruitment of axis inhibition protein (AXIN1/2) and Dishevelled (DVL), which can inhibit the β-catenin destruction complex (consisting of AXIN, GSK3β, CK1, and APC) (233). The result is that unphosphorylated β-catenin is accumulated and then translocates to the nucleus, where it interacts with T cell-specific factor (TCF)/the lymphoid enhancer factor (LEF) family and recruits co-activators, including CBP/p300 and Bcl-9, thereby activating the expression of WNT target genes such as c-Myc, cyclin D1, and CDKN1A. In the absence of WNT ligands, the signaling pathway is OFF, and the destruction complex phosphorylates β-catenin, leading to ubiquitin–proteasomal degradation (235). Moreover, some antagonists, such as dickkopf-1 (DKK1), secreted Frizzled-related proteins (SFRPs), and WNT modulator in surface ectoderm (WISE), are extracellular modulators of Wnt signaling, inhibiting signaling at the ligand or receptor levels (236).

The roles of the canonical Wnt signaling pathway during follicle formation and maintenance have been widely studied (237). One study has verified the involvement of Wnt/β-catenin signaling in granulosa cell apoptosis in North Chinese women with PCOS. In this investigation, transcriptional levels of WNT1, WNT3, and WNT4 were found to be higher in the PCOS group, whereas levels of SFRP4 were lower. Moreover, survivin and BMP4 (downstream survival-related factors) and β-catenin were dramatically decreased. The level of Bax, an apoptosis-related protein, was significantly higher in the PCOS group than in the control group (238). However, these findings were inconsistent with research by Bicer. In this study, serum SFRP4 levels were found to be elevated in women with PCOS; more specifically, the higher a woman’s level of SFRP4, the greater her risk of developing PCOS (239). As an inflammatory adipocytokine, SFRP4 is associated with the onset of diabetes, both type 1 and type 2. It can decrease the influx of Ca^2+^ into β pancreatic cells, thereby suppressing the movement of insulin vesicles toward the membrane and impeding insulin release (240). The dynamic changes occurring during culture *in vitro* and differentiation of GCs could be a reasonable explanation (241). In rat models of PCOS, miR-324-3p inhibits the proliferation of and promotes apoptosis of ovarian granulosa cells by directly targeting WNT2B (242). Similarly, Sanchez and colleagues have also suggested the involvement of the WNT/β-catenin signaling pathway in the luteinized granulosa cell atresia observed in women with endometriosis (243). Another study has investigated the expression profiles of Wnt/β-catenin signaling genes in human oocytes, finding that the expression of WNT1 and GSK3β genes was elevated in PCOS patients, while there was no significant difference between the PCOS group and the control group in terms of APC and β-catenin expression levels. Interestingly, this study did not detect the expression of AXIN2, FZD4, TCF4, WNT5A, WNT3, WNT4, or WNT7A genes in the ovaries of either PCOS patients or healthy women (57). One study has identified the role of FZD3e in FSH-mediated estrogen production (244). Finally, significantly increased FZD3 expression in the cumulus cells of PCOS patients has been found to lead to over-activation of signaling and subsequently more production of stable β-catenin, thus disrupting the recruitment of CYP19A1 promoter (245).

#### 6.8.2 β-catenin-independent signaling

Similar to Wnt/β-catenin signaling, the β-catenin-independent pathway is activated by WNT ligands (such as WNT11 and WNT5A) binding to FZD receptors and ROR1/2 or RYK co-receptors, resulting in activation of downstream effectors (246). One study has observed proinflammatory effects of wnt5a in the GCs of PCOS patients’ ovaries via an interaction between the noncanonical-Ror2 and PI3K/AKT/NF-κB signaling pathways (247). SFRP5 can combine with WNT5a to inhibit the inflammatory process (248). Moreover, inhibition of the NF-κB pathway may disrupt the activation of WNT5A signaling components ROR2 and JNK (249). Similar effects have been thoroughly demonstrated in many studies. For example, WNT-5A/B ligand expression has been found to be increased in the lung fibroblasts of COPD patients, inducing the production of pro-inflammatory cytokines IL-6 and CXCL8 through noncanonical FZD2 signaling (250). The WNT5A ligand is not limited to the noncanonical signaling pathway. Studies have indicated that a switch from canonical to noncanonical WNT signaling can occur. WNT5A can inhibit canonical Wnt signaling activity by competing with the receptor complex, thereby suppressing the secretion of steroid hormones (251, 252).

#### 6.8.3 Crosstalk with Klotho

The Klotho gene is widely recognized to be involved in the anti-aging process in mammals, and under-expression of Klotho is linked to many diseases, including hypertension, diabetes, and chronic kidney disease. The Klotho family includes α-, β-, and γ-Klotho isoforms, which are highly expressed in the kidney but are also found in other tissues such as the liver and AT. Membrane Klotho serves as a co-receptor for many fibroblast growth factors (FGFs) (253). Although exogenous supplementation of Klotho represents a promising treatment for diverse disorders, there is also growing evidence indicating that the over-expression of Klotho and PCOS are closely interrelated. One study measured circulating FGF19, FGF21, and β-Klotho levels in both in PCOS patients and healthy women, finding that β-Klotho levels were significantly higher in PCOS patients; this can be regarded as a strong indicator of PCOS diagnosis (254). In Klotho knock-out mice, the expression level of Wnt1 is increased and the initially inhibited IRS/Akt pathway is restored (255). As mentioned above, these two signaling pathways are both negatively associated with GCs apoptosis. Furthermore, the increased Klotho expression observed in PCOS patients is accompanied by hyperandrogenism. Mechanistically, androgen recruits AR through indirect binding to Klotho promoters at the transcriptional level (256). Meanwhile, other studies have verified that Klotho is expressed in the hypothalamus and the pituitary gland, regulating GH secretion. Of interest, Klotho expression is relatively low in premature ovarian failure (POF), and this upregulates the TGF-β/Smads signaling pathway, which inhibits autophagy. As a consequence, excess ROS disrupts the maturation of oocytes. Hence, it is speculated that the complicated roles played by Klotho are dependent on the signaling pathways in which it participates (257).

#### 6.8.4 Therapeutic strategies

Administration of metformin reverses the low levels of SFRP-5 observed in PCOS patients, and this is expected to be a sensitive indicator for PCOS diagnosis (258). It has reported that CangFu Daotan Decoction normalizes aberrant metabolic features in a rat model of PCOS by suppressing the expression of WNT1 and β-Catenin via modification of m6A methylation (259). Many small molecules or antibody medications have also been found to function as Wnt signaling cascade inhibitors, such as vantictumab (OMP-18R5) and BMD4503-2, which are inhibitors of the Wnt receptor complex; however, development of these focuses primarily on tumor therapy (234, 260). It is believed that, in the near future, more therapeutic strategies targeting Wnt pathway signaling will be developed for PCOS.

### 6.9 The Notch signaling pathway

The Notch pathway is a highly conserved signaling system. In mammals, it has five ligands (JAG1, JAG2, DLL1, DLL3, and DLL4) and four receptors (NOTCH1, NOTCH2, NOTCH3, and NOTCH4). It exerts physiological effects on the phenotype and functional differentiation of vascular endothelial cells. Recent work has demonstrated that endoplasmic reticulum (ER) stress activates Notch signaling in PCOS, particularly Notch2; ultimately, expression of the downstream proteins Hey2 and Hes1 is increased (261).

#### 6.9.1 Angiogenesis

Angiogenesis in the ovary is critical for follicular growth, ovulation, and regression of the corpus luteum (262). Several abnormalities in ovarian angiogenesis have been identified in women with PCOS, including increased ovarian vascularization and blood flow. The dysregulation of some angiogenic factors, such as vascular endothelial growth factor (VEGF), platelet-derived growth factor (PDGF), transforming growth factor-β (TGFβ), and basic fibroblast growth factor (bFGF), may be partially responsible for ovulation and aberrant cysts in PCOS (263). The VEGF family was the first and is now the most widely studied set of angiogenic factors in the ovaries. Studies have shown that VEGF is positively correlated with ovarian blood flow, and there is an increase in the density of blood vessels in the cortical stroma of the ovaries in PCOS. In contrast, notch ligand DLL4, mainly expressed on the endothelial cells, is a negative regulator of VEGF-mediated microvascular growth and branching via the prevention of excessive branching (264). Using an anti-Dll4 monoclonal antibody *in vivo*, Fraser and colleagues showed that this resulted in increased luteal angiogenesis and microvascular density; however, these corpora lutea were less functional and regressed earlier (265). Integrated in silico analysis has identified the Notch pathway as being associated with angiogenesis in PCOS, while the PI3K/Akt signaling pathway is the most enriched pathway (266).

#### 6.9.2 MicroRNAs

Previous studies have indicated that certain miRNAs, such as let-7a, miR-221/miR-222, and miR-92b, are involved in follicular development and follicular atresia. Xu et al. identified miR-483-5p as the regulator of Notch3 expression in human cumulus GC (267). Moreover, LINC00173 has been reported to upregulate JAG1 expression and to be involved in the development of PCOS via downregulation of miR-124-3p (268). Considering the easy access to some miRNAs from blood or fluid samples, it is possible that miRNAs could be used as effective tools for PCOS diagnosis at a very early stage (269).

#### 6.9.3 Steroidogenesis

Research has confirmed that the Notch pathway is also essential for steroid hormone synthesis. Studies have found that, when Notch signaling is inhibited in small preantral follicles, the expression of genes in the steroid biosynthetic pathway is upregulated (270). This may have the ability to regulate GATA4-dependent promoters through the notch downstream effectors HEY1, HEY2, and HEYL. GATA4 is a crucial transcription factor for steroidogenesis (271, 272).

#### 6.9.4 Therapeutic strategies

Notch proteins and ligands are abundant in the hippocampus. It has been found that Notch regulates the functions of learning and memory formation. Disruption of Notch signaling has been implicated as a cause of several human diseases, for example, Alzheimer’s disease (AD) (273). The ligand DLL1 has been shown to be upregulated in the brains of AD patients. Liraglutide (LIR) treatment ameliorates cognitive memory impairment in rats with PCOS via downregulation of Notch overexpression, with the mechanism taking the form of increased acetylcholine levels and decreased Aβ aggregation. Additionally, LIR exerts an anti-inflammatory effect through repression of the activation of the NF-κB pathway (274).

### 6.10 The TGF-β/Smads signaling pathway

The transforming growth factor β (TGF-β) family is crucial for tissue renewal and homeostasis. Its members include molecules such as growth and differentiation factors (GDFs), bone morphogenetic proteins (BMPs), activins, TGF-βs (TGF-β1,2,3), and anti-Müllerian hormone (AMH) (275); their roles in the regulation of ovarian functions have been verified (276). The ligands of the TGF-β family exert their effects by binding to type I or type II receptors (277). In the ligand–receptor complex, type I receptors are phosphorylated by type II receptors, and in turn phosphorylate the R-Smads (Smad2 and Smad3). Subsequently, the activated R-Smads dissociate from the type I receptors and form a complex with co-Smad (Smad4). The complex then translocates to the nucleus, where it regulates the transcription of target genes. In contrast, I-Smads (Smad6 and Smad7), which inhibit the interactions between type I receptors and R-Smads, function as negative regulators of Smad-mediated signaling (278). Studies have indicated that SMAD3, SMAD4, and SMAD5 have close relationships with apoptosis of granulosa cells, the main mechanism underlying follicular atresia (279, 280). TGF-β1 also inhibits the activity of P450 aromatase, an enzyme that converts androgen to estrogen (240, 281).

#### 6.10.1 Fibrosis of the ovary

TGF‐β has three isoforms (TGF‐β1, β2, and β3), with TGF‐β1 being the most common. Many studies have indicated that disruption of the TGF-β1/Smads signaling pathway plays an important role in tissue fibrosis, including renal fibrosis, cardiac fibrosis, and hepatic fibrosis. The mechanism may be due to the transformation of fibroblasts into myofibroblasts, the excessive synthesis of extracellular matrix (ECM), and the inhibition of ECM degradation caused by activation of the TGF-β1/Smads signaling pathway. Moreover, many downstream proteins of TGF-β, such as tissue inhibitor of metalloproteinases (TIMPs), matrix metalloproteinases (MMPs), α-smooth muscle actin (α-SMA), and connective tissue growth factor (CTGF), contribute to organ fibrosis (276). To date, however, few studies have focused on ovarian fibrosis. Recent studies have linked this condition to many ovarian diseases, including PCOS and POI (282). The common features of ovarian fibrosis in PCOS patients are a thickened tunica albuginea, due to increased collagen deposition, and increased cortical stroma. Therefore, the aberrant theca cells produce more steroid hormones (283). TGF-β1 also inhibits the activity of P450 aromatase, an enzyme that converts androgen to estrogen (240, 281). TGF-β1 gene polymorphisms are associated with the development of PCOS and characteristics of women with PCOS in the Korean and Han Chinese population. A study has confirmed that ER stress, a potential determinant of pro-fibrotic remodeling during tissue fibrosis, is activated in the granulosa cells of the ovary in PCOS via induction of TGF-β1 expression (284). Over-activation of the NLRP3 inflammasome, induced by excess androgen, results in significantly increased levels of fibrotic factors, such as TGF-β, CTGF, α-SMA, β-catenin, and the environment becomes more collagenous, with increased expression of collagen I and collagen IV (107). However, it remains unclear whether ovarian fibrosis is the cause of PCOS or the result.

#### 6.10.2 Fibrillin and follistatin

The Fibrillin 3 gene (allele 8 of D19S884) appears to be a susceptibility gene for PCOS and is associated with impaired glucose homeostasis (276). Fibrillin 3 regulates the bioactivity of TGF-β by binding to the TGF-β superfamily ligands. Hence, some variants of the Fibrillin gene are thought to alter the normal function of TGF-β signaling and contribute to the pathogenesis of PCOS (285). Elevated follistatin levels have been detected in PCOS patients, regardless of weight. As this is an activin-binding protein, increased follistatin levels result in the neutralization of more activin, which is capable of stimulating FSH secretion. Accordingly, this leads to more androgen production by theca cells and disrupts follicular development (286, 287).

#### 6.10.3 Growth differentiation factors

As mentioned above, GDF8 also belongs to the TGF-β superfamily and plays crucial roles in the regulation of folliculogenesis, in steroidogenesis, and in luteal function (288). As an intra-ovarian factor, a number of studies have linked dysregulated GDF8 to many types of reproductive disorders, such as ovarian hyperstimulation syndrome (OHSS) and PCOS. GDF-8 expression levels in follicular fluid and in serum tend to be elevated in PCOS patients, although it is noteworthy that this effect is only observed in obese PCOS patients (289). Moreover, elevated GDF8 levels in women with PCOS often function as a predictor of poorer pregnancy outcomes when women are undergoing IVF treatment (290). One study has revealed that GDF8 is the culprit for the abnormal glucose metabolism observed in PCOS as a result of its stimulation of the expression of SERPINE1 through the ALK5-mediated SMAD2/3-SMAD4 signaling pathway. The SERPINE1 gene encodes plasminogen activator inhibitor 1 (PAI-1), leading to hypofibrinolysis, and is closely associated with diabetes and IR (291, 292). Intriguingly, unlike GDF-8, there is no obvious difference between normal and polycystic ovaries in the expression of GDF11, which has a similar molecular structure and function to GDF8 (293).

#### 6.10.4 Therapeutic strategies

##### 6.10.4.1 Small molecules

One study has proven that a potent ALK5 inhibitor, namely SB431542, can inhibit related molecules, such as TGF-β, Smad3, Smad2, and a-SMA, and upregulates anti-fibrotic factor MMP2 in rats with DHEA-induced PCOS, thereby mitigating ovarian fibrosis via the TGF-β/Smads signaling pathway (283). Moreover, its value in improving ovarian morphology in PCOS has been verified (291). Given the high levels of GDF8 and GDF15 observed in patients with PCOS, weight management measures, such as exercises, training, and a ketogenic diet, appear to be a good option (294–296).

#### 6.10.5 Drugs

##### 6.10.5.1 Rosiglitazone

Rosiglitazone, a PPAR-γ agonist, may alleviate ovarian fibrosis by suppressing the transduction of TGF-β1 and lowering CTGF levels in a rat model of PCOS (282). PPAR-γ has been found to inhibit nuclear translocation of the transcription factor NF-κB and activator protein-1 (AP-1), thus serving anti-inflammatory and anti-fibrotic roles (297).

##### 6.10.5.2 Sitagliptin

The dipeptidyl peptidase-4 (DPP4) inhibitor sitagliptin is widely used to treat type 2 diabetes, but researchers have recently found that it also has a therapeutic effect in renal and hepatic fibrosis. A study has demonstrated that sitagliptin can suppress ovarian fibrosis in rats with PCOS through downregulation of the TGF‐β1/Smad2/3 signaling pathway (298).

#### 6.10.6 Phytochemicals

##### 6.10.6.1 Proanthocyanidins

PCs, derived from many dark-green leafy vegetables, improve ovarian fibrosis in mice with PCOS through regulation of serum hormone levels, inhibiting oxidative stress and suppressing activation of the TGF-β1/Smads signaling pathway (299).

##### 6.10.6.2 Paeoniflorin

PAE, a major active component of Paeonia lactiflora Pallas, has potent anti-inflammatory and immune-regulatory effects (300). Zhou et al. have shown that PAE may reduce the expression of TGF-β1 and Smad3, while increasing the expression of Smad7 and MMP2 (a negative regulator of the TGF-β1/Smads signaling pathway), especially at a high dose of PAE, in rat models (301). These findings are consistent with previous studies on the effects of PAE in the treatment of liver fibrosis, myocardial fibrosis, and lung fibrosis.

##### 6.10.6.3 Rhamnocitrin

Rha has also been discovered to downregulate the TGF-β1/Smads signaling pathway and to suppress NF-κB through activation of PPAR-γ (302, 303).

##### 6.10.6.4 Resveratrol

Regulation of the TGF-β signaling pathway and ROS are interconnected. ROS can stimulate the TGFβ ligand and promote the expression of fibroblast TGF-β; in turn, TGF-β induces an increase in ROS in certain tumor cells (304). Resveratrol exerts anti-fibrotic and anti-apoptotic effects through inhibition of p66Shc-mediated ROS production and expression of fibrotic factors (305, 306).

### 6.11 The Hedgehog signaling pathway

Hedgehog (Hh) signaling has been implicated in a regulatory role in the developmental processes of both embryo and adult tissues. Abnormal activation of Hh signaling has been linked to numerous types of cancer. The mammalian hedgehog family consists of three ligands [sonic hedgehog (SHH), desert hedgehog (DHH), and Indian hedgehog (IHH)], two receptors (PTCH1 and PTCH2), and the seven-transmembrane signal transducer protein Smoothened (SMO). If there is no ligand, PTCH1 or PTCH2 inhibits the activity of SMO. Once the ligand binds to the receptor, SMO inhibition is lifted, leading to the activation of downstream transcription factors, namely glioma-associated oncogene homologs (Gli1, Gli2, and Gli3), in the nucleus (307). Many studies have demonstrated its impact on follicle growth and the proliferation of theca and granulosa cells (308). A lack of Dhh/Ihh in the ovaries causes theca cell loss, disrupted steroid production, and failure to form the corpora lutea (309). According to pathway analysis based on genome-wide methylation profiling in the granulosa lutein cells of PCOS patients, the Hedgehog pathway is associated with the development of PCOS (310).

#### 6.11.1 Steroidogenesis

One of the main characteristics of PCOS patients is HA, and the ovarian theca cells are thought to be one of the major sources of excess androgen in PCOS patients (311). Liu et al. have demonstrated that establishment of a theca cell lineage requires both granulosa cells and oocytes, with involvement through multicellular interactions via GDF9 and Hh signaling (309). Interestingly, HH ligands (DHH and IHH) are expressed in the granulosa cells of primary-to-antral-stage follicles, whereas the downstream effector Gli1 is found in the theca layer (312). Moreover, expression of Srd5a3 and Cyp17a1, which are steroidogenesis-related genes, has been found to be decreased in Ihh single-knockout mice. This implies a role for Ihh in the regulation of steroidogenesis in the ovary, and the same study also proved that Ihh has greater influence on the activation of the Hedgehog signaling pathway (313).

#### 6.11.2 Follicular growth and anovulation

In the absence of Dhh and Ihh, the theca cell layer fails to form and preantral follicles are unable to develop. Li observed higher levels of Ihh and Ptch2 in PCOS patients based on RT-PCR analysis when compared to a control group, indicating over-expression of the Hh signaling pathway in PCOS patients. This is also related to the aberrant follicular growth observed in PCOS patients (314). Yi et al. have demonstrated that overactivation of the Hedgehog signaling pathway in the ovary inhibits ovulation. In their experiment, transgenic mice expressing a dominant active allele of the signal transducer Smoothened (SmoM2) were able to prevent the suppression of its activity by PTCH, thus leading to sustaining activation of Hedgehog signaling. The Amhr2cre/+SmoM2 mice exhibited extremely reduced ovulation. This may be attributable to impaired muscle cells or contractile cells in the theca layer, which are very important for ovulation (315, 316). Another study has demonstrated that the anovulatory follicles of mhr2cre/+SmoM2 mutant mice are caused by denser capillaries in the ovarian cortex and a lack of vascular smooth muscle in the theca cells of such mice (317). Therefore, it is very likely that disrupted SH signaling contributes to the development of anovulation in PCOS (311). High levels of GTPase immunity-associated protein (GIMAP) 7 expression is observed in the GCs of PCOS patients. In contrast, silencing of the GIMAP7 gene protects GCs from apoptosis and OS via upregulation of the SHH signaling pathway. The expression of downstream components SHH and SMO is increased, along with increased levels of the antioxidants SOD and GSH (318).

#### 6.11.3 Therapeutic strategies

The Hh signaling pathway inhibitor cyclopamine can decrease apoptosis of ovarian granulosa cells in PCOS (314). Conversely, another study has revealed that administration of cyclopamine partially counteracts the protective effects of GIMAP7 knockout on rats with PCOS with increased ROS levels (318). Further studies focusing on the impact of different Hh ligands on PCOS development are warranted. Moreover, exposure to certain chemicals, such as acetazolamide and itraconazole, as well as maternal smoking, can disrupt HH signaling, thereby interfering with the development of follicles and contributed to infertility to some extent (319). More attention needs to be paid to the avoidance of exposure to these substances.

## 7 Conclusion and future directions

**Figure 7:**
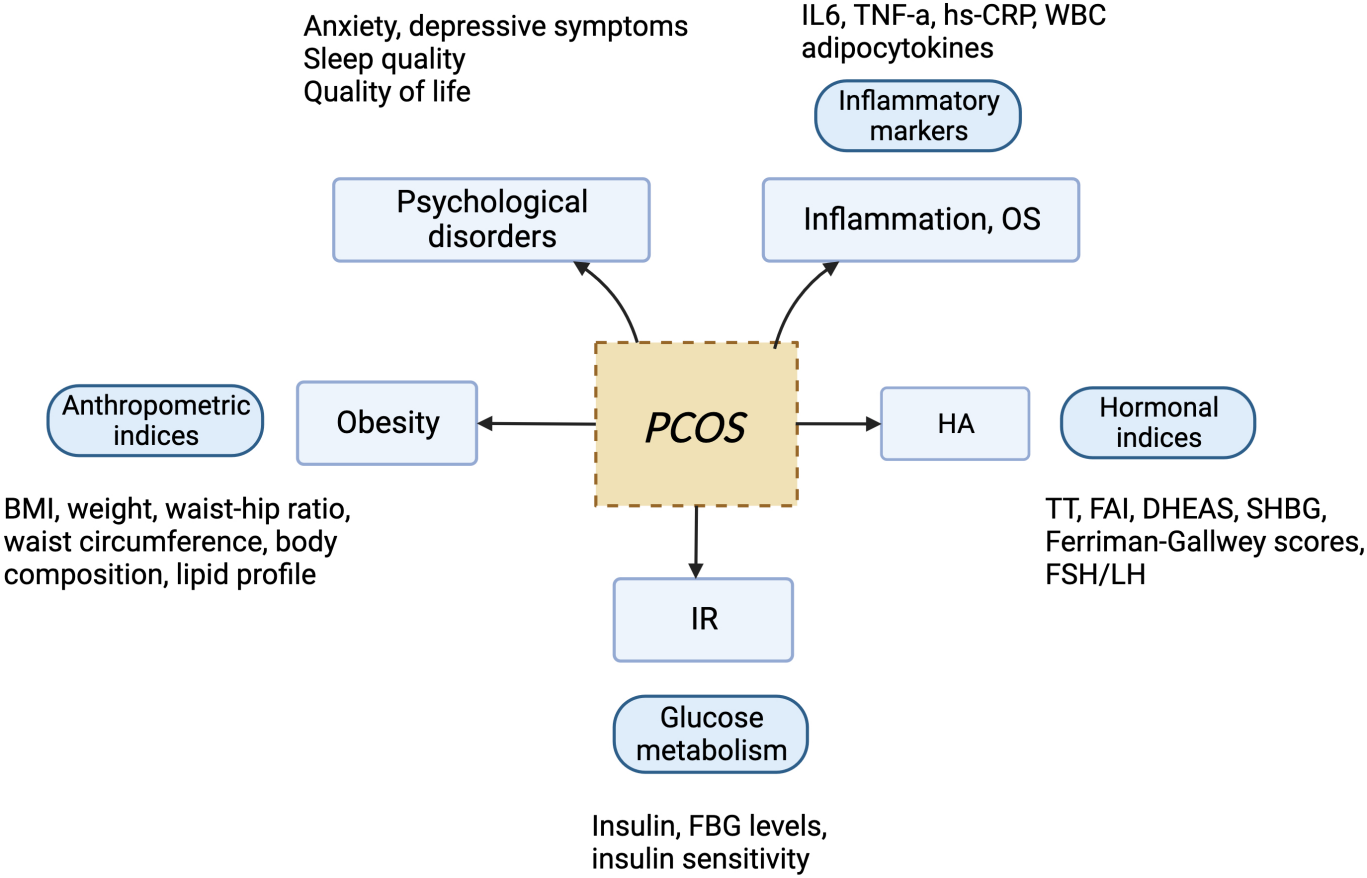
The effects of drugs, dietary supplements, and non-pharmacological treatments on the main health outcomes associated with PCOS. HA, hyperandrogenism; IR, insulin resistance; TT, total testosterone; SHBG, sex hormone-binding globulin; FAI, free androgen index; FBG, fasting blood glucose; BMI, body mass index.

As it is an intricately complex disorder, multiple signaling pathways and components are involved in the pathophysiological processes of PCOS, and intriguing interactions occur between each signaling cascade. These pathways collectively regulate metabolic and ovarian functions, including folliculogenesis, steroidogenesis, angiogenesis, oocyte maturation, and ovulation. Dysregulation of these pathways contributes jointly to the pathological states involved in PCOS, such as IR, HA, OS, and inflammation (**Figure 7**). Moreover, some molecules (for instance, SFRP-5, β-Klotho, leptins, and certain miRNAs) are considered to be promising diagnostic biomarkers for PCOS. We have outlined the relevant signal transduction pathways involved in the development of PCOS as well as the crosstalk between them. Inspiringly, several bioactive compounds derived from herbs and TCM have exhibited unique advantages in PCOS treatment in recent years. In **Table 1**, we list current pharmacological approaches targeting the signaling pathways involved in order to reverse PCOS symptoms and restore ovarian functions in animal models or at the cellular level. However, until now, the options for agents and supplements to treat this condition have been limited, with most such treatments being off-label. Fortunately, the efficacy of some of these interventions has been evaluated in clinical trials, which have shown promising results in the management of PCOS. In **Table 2**, we present an investigation and summary of glucose-lowering agents, dietary supplements (including minerals, e.g., chromium, zinc, calcium, and magnesium; vitamins; and other nutrients, e.g., herbs and probiotic and polyphenolic compounds), and in **Table 3**, we list some non-pharmacological treatments for PCOS in view of these clinical trials.

**Table 1:** Summary of pharmacological therapies targeting signaling pathways involved in PCOS pathogenesis in animal models.

Treatment	Target signaling pathway	Reference
Plumbagin	PI3K/Akt/mTOR NLRP3 inflammasome	PMID: 33029296 PMID: 36463191
Cangfu Daotan decoction	IGF-1/PI3K/Akt ASK1/JNK(MAPK) Wnt/β-catenin	PMID: 33299379 PMID: 36465612 PMID: 36822223
Liuwei Dihuang pills	PI3K/Akt	PMID: 31185267
Luteolin	PI3K/AKT Nrf2-HO	PMID: 33900851
Heqi San	PI3K/AKT	PMID: 29020999
Berberine	PI3K/Akt MAPK	PMID: 33709493 PMID: 31778716
Sesame oil (SO)	PI3K/PKA MAPK/ERK2	PMID: 36810739
Lidocaine	PI3K/AKT/mTOR	PMID: 35464164
Guizhi Fuling Wan	PI3K/AKT/mTOR	PMID: 33460753
Hehuan Yin decoction	PI3K/Akt	PMID: 35121050
Shouwu Jiangqi decoction	IRS/PI3K/Akt	PMID: 26179926
Bu-Shen-Tian-Jing formula	PI3K/AKT Sirtuin 3/p38MAPK	PMID:36610149
Humanin	IRS1/PI3K/Akt Keap1/Nrf2	PMID: 33693742 PMID: 33337472
Growth hormone	PI3K/Akt	PMID: 33287836
Liquiritin	PI3K/AKT	PMID: 35733703
Melatonin	PI3K/Akt	PMID: 33989172 PMID: 34953312 PMID: 35228471
Eucommia ulmoides Oliv. leaves (TFEL)	PI3K/Akt	PMID: 33617969
Chemerin	PI3K/AKT/mTOR	PMID: 34816741
Selenium nanoparticles and metformin	PI3K/Akt	PMID: 36661246
Crocus sativus (saffron) petal extract	NF- κB	PMID: 34480994
Soy isoflavones	NF- κB	PMID: 34546841
Apigenin	NF- κB	PMID: 31888395
Cryptotanshinone (CRY)	NF- κB MAPK/ERK	PMID: 35419170 PMID: 32901834
Shaoyao-Gancao decoction (SGD)	TLR4/NF-κB	PMID: 30573529 PMID: 34054541
Quercetin	TLR4/NF-κB	PMID: 27634381
Bu Shen Hua Zhuo formula	LPS/TLR4/NF- κB	PMID: 36017323
Sulforaphane (SFN)	Keap1/Nrf2	PMID: 34939763 PMID: 30032437
Linagliptin and/or I3C	Keap1/Nrf2	PMID: 28619389
Genistein	Keap1/Nrf2	PMID: 34374402
Qi Gong Wan	Nrf2/ HO-1/Cyp1b1	PMID: 36273747
Astaxanthin	Nrf2-HO-1	PMID: 35237893
Diacerein (DIA)	Keap1/Nrf2/HO-1 IL-1β/NFκB	PMID: 35367769
Baicalin	AMPK	PMID: 31722718
Fisetin	AMPK	PMID: 34298098
Vitamin D3	AMPK MAPK	PMID: 29676471 PMID: 32048305
Quercetin	AMPK/SIRT1	PMID: 29105398 PMID: 33798596 PMID: 35889348
Naringenin	AMPK/SIRT1	PMID: 35724506
N1-methylnicotinamide	AMPK	PMID: 32298659
myo-inositol	IL6/STAT3	PMID: 32045334
Nervilia Fordii (total flavonoids)	JAK2/STAT3	PMID: 30463907
Troxerutin	JAK1/STAT3	PMID: 36103628
Resveratrol	TGF-β/Smads	PMID: 32066482
Irpex lacteus polysaccharide	TGF-β1/Smad	PMID: 37554783
Rhamnocitrin (Rha)	TGF-β/Smads	PMID: 35965823 PMID: 35663203
Paeoniflorin (PAE)	TGF-β/Smads	PMID: 33638949
Proanthocyanidins (PCs)	TGF-β/Smads	PMID: 33818798

**Table 2:** Various clinical trials of drugs and supplements for PCOS.

Treatment	Description of main intervention	Outcomes	Reference or registration number
Flaxseed supplementation	Women with PCOS received flaxseed powder (30g/day) plus lifestyle modification or only lifestyle modification for 12 weeks.	Significant reduction in body weight and in insulin, TG, hs-CRP, and IL-6 levels.	IRCT20120704010181N11
L-carnitine supplementation	Overweight/obese women with PCOS received 1000 mg/day L-carnitine or placebo (1000 mg starch) for 12 weeks.	Significant improvement in insulin, with no effect on SHBG or lipid profile.	IRCT20191016045131N1
Acetyl-L-carnitine (ALC)	Women with PCOS received a combination of metformin, pioglitazone, and ALC (500 mg, 15 mg, and 1500 mg) twice daily or metformin plus pioglitazone and placebo for 12 weeks.	Significant improvements in adiponectin, T levels, IR, menstrual cycle, and body circumference.	NCT04113889
Curcumin	Women with PCOS received 500 mg curcumin three times daily or placebo for 12 weeks.	Significant reduction in FPG and DHEA levels.	PMID: 33137599
Resveratrol	Women with PCOS received 1,500 mg/day or placebo for 12 weeks.	DHEAS and fasting insulin levels decreased; no changes in gonadotropins, lipid profile, or inflammation markers.	NCT01720459
Resveratrol and myoinositol combination therapy	Obese, oligo-anovulatory women with PCOS received a combination of metformin and pioglitazone (500 mg and 15 mg) twice daily, or a combination of resveratrol and myoinositol (1000 mg and 1000 mg) twice daily for 12 weeks.	Significant changes in T, adiponectin, gonadotropins, BMI, and menstrual regularity.	NCT04867252
Coenzyme Q10	Women with PCOS took 100mg/d CoQ10 or placebo for 12 weeks.	Significant improvements in glucose metabolism (FPG, insulin).	PMID: 27911471
Coadministration of vitamin D and omega-3 fatty acid	Women with PCOS took either 50,000 IU vitamin D every 2 weeks plus 2,000 mg/d omega-3 fatty acid from fish oil or placebo for 12 weeks.	Significant improvements in TT, CRP, IL-1, VEGF, and malondialdehyde (MDA) levels; anxiety; and depressive symptoms.	PMID: 29859385
Melatonin and/or magnesium supplementation	PCOS women took magnesium, melatonin, magnesium plus melatonin, or placebo for 8 weeks.	Co-supplementation resulted in significant improvements in TT, IR, lipid profile, and sleep quality.	IRCT20191130045556N1
Probiotics	Women with PCOS underwent probiotic intervention, placebo, or metformin treatment for 6 months.	Not yet complete.	NCT04593459
Oral contraception (OCP)	Women with PCOS underwent treatment with continuous use of OCP, intensive lifestyle modification, or a combination of both.	Significant changes in reproductive/metabolic hormones in the OCP group.	NCT00704912
Atorvastatin	Women with PCOS received 20mg/d atorvastatin or placebo for 6 months.	DHEAS, CRP, LDL, and TG levels decreased; no change in serum T levels or insulin sensitivity impairment.	NCT01072097
Canagliflozin (CANA) and metformin combination therapy	Overweight or obese non-diabetic women with PCOS received CANA (100 mg/d) plus MET (1000 mg twice daily) or MET only (1000 mg twice daily) for 3 months.	Significant changes in TT level and area under the curve for glucose.	NCT04973891
Spironolactone and metformin combination therapy	Women with PCOS received metformin (1000 mg/d), spironolactone (50mg/d), or a combination of the two drugs for 6 months.	Decreased TT, insulin levels, and Ferriman-Gallwey scores, with no significant change in body weight, BMI, waist–hip ratio, or blood pressure.	PMID: 23846820
Berberine (BBR)	Women with PCOS received BBR+ cyproterone acetate (CPA), MET+CPA, or placebo+CPA for 3 months.	Decrease in waist circumference and waist-to-hip ratio, total cholesterol, TG, and LDLC; increase in HDLC and SHBG.	PMID: 22019891
Liraglutide	Women with PCOS received LIRA (3 mg/d) or placebo with lifestyle intervention for 32 weeks.	Significant changes in FAI level and body weight.	NCT03480022

**Table 3:** Clinical trials of non-pharmacological treatments for PCOS.

Treatment	Description/main intervention	Outcomes/results	Registration number
Time-restricted feeding (TRF)	Women with POCS with anovulation underwent a 1-week baseline weight stabilization period and a 5-week TRF period.	Significant weight loss; improvements in menstruation, HA, IR, and chronic inflammation.	NCT04580433
Ketogenic diet	Overweight women adopted a ketogenic Mediterranean diet with phytoextracts for 12 weeks.	Significant weight loss; changes in insulin, DHEAS, LDL, TG, and total cholesterol levels, with increased HDL levels.	NCT04163120
Pulse-based diet	Women with PCOS adopted a pulse-based diet or therapeutic lifestyle changes (TLC) for 12 weeks.	Significant changes in HDL, total cholesterol levels, and diastolic blood pressure.	NCT01288638.
Acupuncture	Women with PCOS received true acupuncture plus placebo, metformin (0.5 g three times daily) plus sham acupuncture, or sham acupuncture plus placebo for 4 months.	The true acupuncture group did not improve in insulin sensitivity as effectively as improvements induced by metformin treatment in women with PCOS and IR.	NCT02491333
Cognitive–behavioral therapy (CBT)	Women with PCOS received cognitive–behavioral therapy (8 sessions of 60–90 min weekly) in groups of 5 to 7 people.	Improvements in depressive symptoms, anxiety symptoms, and quality of life.	IRCT20110826007418N7 NCT01899001
High-intensity interval training	Women with PCOS underwent high-intensity interval training (HIIT) or moderate-intensity continuous training (MICT) 3 times per week for 12 weeks.	Significant improvements in menstrual cyclicity and SHBG levels.	ACTRN12615000242527

Since little is known about this disease, many women suffer from infertility, hirsutism, irregular menstrual periods, psychological disorders, and long-term complications brought about by PCOS throughout their lives. More in-depth research and greater attention to PCOS are required in order to solve the mystery of this condition.

## Author contributions

KW wrote the original manuscript and completed the figures and tables. YL helped write and revise this paper. All authors contributed to the article and approved the submitted version.
